# Modeling inducible neuropathologies of the retina with differential phenotypes in organoids

**DOI:** 10.3389/fncel.2023.1106287

**Published:** 2023-05-05

**Authors:** Manuela Völkner, Felix Wagner, Thomas Kurth, Alex M. Sykes, Claudia Del Toro Runzer, Lynn J. A. Ebner, Cagri Kavak, Vasileia Ismini Alexaki, Peter Cimalla, Mirko Mehner, Edmund Koch, Mike O. Karl

**Affiliations:** ^1^Technische Universität Dresden, Center for Regenerative Therapies Dresden (CRTD), Dresden, Germany; ^2^German Center for Neurodegenerative Diseases (DZNE), Dresden, Germany; ^3^Technische Universität Dresden, Center for Molecular and Cellular Bioengineering (CMCB), Technology Platform Core Facility Electron Microscopy and Histology, Dresden, Germany; ^4^Max Planck Institute of Molecular Cell Biology and Genetics, Dresden, Germany; ^5^Technische Universität Dresden, Institute of Clinical Chemistry and Laboratory Medicine, University Clinic Carl Gustav Carus, Dresden, Germany; ^6^Technische Universität Dresden, Carl Gustav Carus Faculty of Medicine, Department of Anesthesiology and Intensive Care Medicine, Clinical Sensoring and Monitoring, Dresden, Germany

**Keywords:** retina, photoreceptor, glia, mouse organoid, pathology modeling, neurodegeneration, neuron, mouse embryonic stem (mES) cells

## Abstract

Neurodegenerative diseases remain incompletely understood and therapies are needed. Stem cell-derived organoid models facilitate fundamental and translational medicine research. However, to which extent differential neuronal and glial pathologic processes can be reproduced in current systems is still unclear. Here, we tested 16 different chemical, physical, and cell functional manipulations in mouse retina organoids to further explore this. Some of the treatments induce differential phenotypes, indicating that organoids are competent to reproduce distinct pathologic processes. Notably, mouse retina organoids even reproduce a complex pathology phenotype with combined photoreceptor neurodegeneration and glial pathologies upon combined (not single) application of HBEGF and TNF, two factors previously associated with neurodegenerative diseases. Pharmacological inhibitors for MAPK signaling completely prevent photoreceptor and glial pathologies, while inhibitors for Rho/ROCK, NFkB, and CDK4 differentially affect them. In conclusion, mouse retina organoids facilitate reproduction of distinct and complex pathologies, mechanistic access, insights for further organoid optimization, and modeling of differential phenotypes for future applications in fundamental and translational medicine research.

## 1. Introduction

Neuropathologies throughout the nervous system may have many different causes, and present with different phenotypes, due to distinct combinations of pathologic processes of neurons, glia, immune cells, like microglia, and others. For example, blindness may be caused by neuropathologies of the retina (Hartong et al., 2006; Fleckenstein et al., 2021), which in vertebrates is composed of six neuronal cell types, structured into three nuclear layers: The light-sensitive cone and rod photoreceptors (PRs) in the outer retina are connected to interneurons (bipolars, amacrines, and horizontals) and supported by radial glia, i.e., Müller glia (MG), in the inner retina, followed by a layer of ganglion neurons that connect to higher brain centers. Due to many different gene mutations, cones, and rods degenerate in both inherited retinal dystrophies (IRDs) and in macular degenerative diseases, particularly the common age-related macular degeneration (AMD). Notably, glia may cause and contribute to neuropathologies with neuroprotective and detrimental functions, summarized as reactive gliosis, which may involve glia proliferation, hypertrophy, and scarring (Bringmann et al., 2009). Retina remodeling, which is a combination of glial scarring and several other changes throughout the retina, occurs secondarily in most diseases at late stages. Notably, AMD and some IRDs present complex pathology phenotypes, combining PR degeneration and remodeling (Sadda et al., 2018; Fleckenstein et al., 2021). Microglia are another cell type in the retina with several functions in the healthy and diseased retina: In particular, they participate in neurodegeneration by producing proinflammatory factors and removing neurons (Akhtar-Schafer et al., 2018; Silverman and Wong, 2018). Neuronal, glial, and remodeling processes, specifically their inducers and drivers, are still insufficiently understood in many pathologies (Hartong et al., 2006; Sancho-Pelluz et al., 2008; Murakami et al., 2013; Fritsche et al., 2014; Sudharsan et al., 2017; Pfeiffer et al., 2020; Fleckenstein et al., 2021; Olivares-Gonzalez et al., 2021): This presents untapped therapeutic targets to prevent and treat vision loss in a broad patient spectrum. So far, there are still no effective retinal therapies (Scholl et al., 2016), and it is also not yet clear how to effectively transfer such therapies to patients. Pluripotent stem cell-derived organoid technologies facilitate research into fundamental and preclinical translational medicine. For example, organoids are advantageous for larger-scale studies and complex multiple manipulations: They might also complement longitudinal 3D live imaging studies of distinct cellular and molecular processes using fluorescent reporters. In contrast, experiments with patient- and animal-derived retinas are limited due to the low number of high-quality postmortem donor retinas and ethical considerations, respectively.

Since the pioneering reports of mouse (MROs) and human retina organoids (HROs) (Eiraku et al., 2011; Meyer et al., 2011; Nakano et al., 2012), more advanced protocols have been developed (Gonzalez-Cordero et al., 2013; Zhong et al., 2014; Volkner et al., 2016, 2022; Chen et al., 2020; Cowan et al., 2020; Decembrini et al., 2020) to improve organoid yield and reduce variance. Similarities but also differences in development, cell composition, and transcriptomics have been described for MROs/HROs compared to primary retina (DiStefano et al., 2018; Brooks et al., 2019; Kaya et al., 2019; Kim et al., 2019; Cowan et al., 2020; Sridhar et al., 2020). However, current organoid systems have not yet been studied systematically, and beyond neonatal stages. Since early organoids do not contain all parts of the *in vivo* retina, recent studies have started to integrate additional cell types. For example, microglia have been introduced to human organoids, since these do not develop within the retinal lineage, but enter during retinogenesis and remain throughout life (Silverman and Wong, 2018). Thus, one advantage of organoids is that pathology modeling can be studied in the absence of several cell types, e.g., vasculature or immune cells. The contribution of different cell types upon their addition at defined timepoints during pathogenesis can also be tested. So far, studies in animal *in vivo* models have depended on the experimental removal of such cells, which may in itself cause a pathology (Wang et al., 2016). Organoids have been used to study development (Capowski et al., 2019; Cowan et al., 2020; Sridhar et al., 2020), gene and cell replacement therapies (Santos-Ferreira et al., 2016; Deng et al., 2018; Kruczek et al., 2021; Volkner et al., 2021b; Wagner et al., 2022), and pathologies. Specifically, developmental disorders and gene mutation-dependent impairments in cell-specific genes and proteins, distinct loss of retinal cells and structures (Bell et al., 2020; Kruczek and Swaroop, 2020; Zhang et al., 2021), experimentally induced PR degeneration (Ito et al., 2017; Koukourakis et al., 2022), and complex phenotypes (Quinn et al., 2019; Volkner et al., 2022) have been reproduced. However, most pathology models so far have been established in HROs, and a few in MROs (Ito et al., 2017; Mookherjee et al., 2018; Kruczek et al., 2021; Volkner et al., 2021a). While HROs are, in the long run, the most promising for preclinical translational research such as patient cell-derived models, and pathologies not completely reproducible in animals, HROs require more time and resources than MROs. It may take more than 200 days to develop HROs, whereas MROs only require about 20 days. MROs therefore provide a useful system to develop models prior to transfer to HROs. Further, any established model, derived pathomechanism, and therapeutic target needs to be validated, but patient samples are scarce so this can be a significant challenge. Thus, MRO models in combination with the matching experimental or genetic animal models might facilitate effective HRO pathology modeling to effectively establish human patient-specific pathology models, study pathomechanisms, and develop therapies. When combined, these might synergize to advance organoid system optimization and applications. However, it is generally still unclear to what extent differential pathology phenotypes with distinct and complex processes can be induced and reproduced in MROs.

Here, we explored pathology modeling by screening 16 different experimentally induced chemical, physical, and cell functional manipulations in an established organoid system (Volkner et al., 2016, 2019, 2021a). Most notably, we found that combined application of HBEGF and TNF (HT) induces a severe pathology in MROs. Based on these data, we recently developed a complex retina pathology model in HROs, reproducing several distinct neuronal and glial pathologic processes from early- to late-stage pathogenesis (Volkner et al., 2022). Here, we established and characterized the HT-induced pathology in MROs, and we show that it can be prevented by pharmacologicals, indicating mechanistic access. Interestingly, the major pathological phenotypes induced by HT are comparable in MROs and HROs, however, pathomechanisms of PR degeneration seems to slightly differ between both system. Taken together, our results show that early post-mitotic MROs are competent to reproduce differential pathology phenotypes with distinct neuronal and glial processes, and our data further support the notion that the HT model might reproduce key processes of IRDs and AMD in patients and animals.

## 2. Materials and methods

### 2.1. Mouse embryonic stem cell and organoid culture

E14TG2a (MMRRC, UC Davis) mouse embryonic stem cells (mESCs) were cultured in mESC medium (DMEM, 15% FBS, 1% pyruvate, 1% NEAA, 1% GlutaMAX, 1% penicillin/streptomycin, and 1 mM 2-mercaptoethanol) supplemented with 10^3^ U/ml LIF and 1 μM PD0325901 on 10 cm tissue-culture plates (BD Falcon). Cells were passaged every 2 days using TrypLE Express (Invitrogen); replated at a density of 1 × 10^6^ cells/plate. Mouse retina organoids (MROs) were generated as previously reported (Volkner et al., 2016, 2019). Briefly, mESCs were dissociated to single cells using TrypLE Express (Invitrogen), 3000 cells/well were plated in 96-well low-adhesion plates (Lipidure Coat, NOF) and cultured (37°C, 20% O_2_) in retinal differentiation medium (RDM: GMEM, 1% penicillin/streptomycin, 1% NEAA, 1% pyruvate, 1.5% KnockOut Serum Replacement, and 1 mM 2-mercaptoethanol). On day (D) 1, 2% Matrigel (growth-factor reduced, BD Biosciences) was added. On D7, organoids were transferred to petri dishes (Greiner Bio-One) and cultured (37°C, 40% O_2_) in DMEM/F12 with GlutaMAX, 1% N-2 and 1% penicillin/streptomycin. On D10, organoids were manually trisected using surgical tweezers (Dumont No. 5) and further cultured in DMEM/F12 with GlutaMAX, 1% N-2 supplement, 1% penicillin/streptomycin, 10% FBS. Synthetic retinoid analog EC23 (0.3 μM) was added from D10 to D14.

### 2.2. MRO challenge screen

To establish organoid pathology models, experimental challenges, including chemical, physical, and cell functional manipulations, were applied acutely or daily, and analyzed after 5 days (see Supplementary Experimental Procedures in Supplementary Table 1).

### 2.3. Tissue preparation and immunohistochemistry

For immunostaining analysis samples were fixed in 4% paraformaldehyde, cryoprotected, and embedded in tissue-freezing medium (Jung). Frozen sections were cut at 12 μm. Immunostaining was performed using standard protocols. See Supplementary Experimental Procedures for further details.

### 2.4. Data analysis and statistics

Samples were imaged on a Zeiss ApoTome2 microscope. Statistical analysis was performed with GraphPad Prism 8 software using 1-way ANOVA (Tukey’s *post-hoc* test) or Student’s unpaired *t*-test. Results were considered significant for *p* < 0.05, and data were plotted as mean ± standard deviation (SD). Standard deviations were computed for total organoid numbers (n) from N experiments. *N* ≥ 3 independent experiments with *n* ≥ 5 organoids per experiment were analyzed for each dataset, unless stated otherwise in the respective figure legend or Supplementary Experimental Procedures. Graphs and schematic illustrations were prepared using GraphPad Prism 8 and Adobe Illustrator CC software, respectively. Images were optimized by making minor changes to contrast, and cropped in Adobe Photoshop CC and arranged using Adobe Illustrator CC.

## 3. Results

### 3.1. Exploring experimentally inducible neuronal damage models in MROs

**FIGURE 1:**
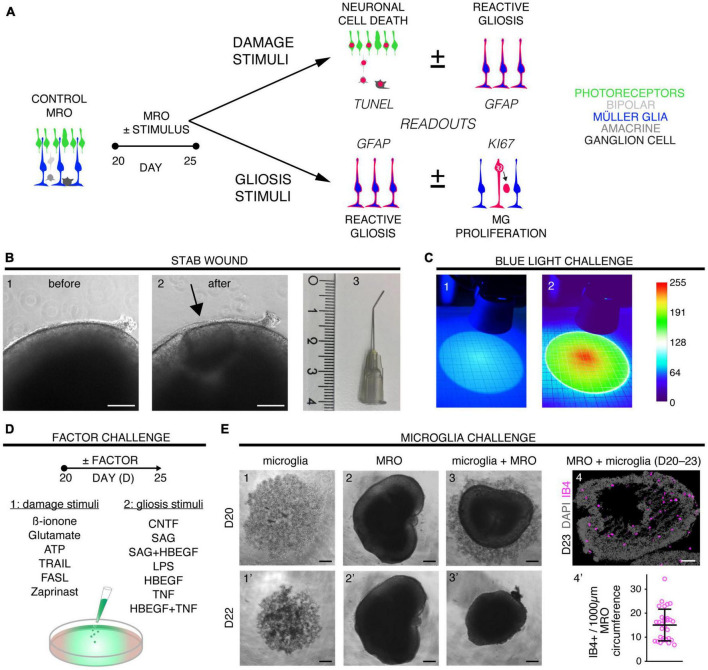
Exploring inducible neuropathologies with differential phenotypes in MROs. **(A)** Schematic of the experimental design for pathology modeling in mouse retina organoids (MROs): 16 challenges, including physical, chemical, and cell functional manipulations, to induce neuronal cell death **(B,C**,D1) or to directly stimulate MG damage responses (D2,**E)**. Drawings illustrate the relevant readouts: Cell death (TUNEL assay), proliferation (KI67), and gliosis (GFAP) were quantified on immunostained serial MRO sections. **(B)** To induce a stab wound in mouse retina organoids, a 27G blunt-end needle was used to stab the MRO retinal epithelia. Representative phase-contrast images of the MRO retinal epithelium before (B1,B2) after wounding (scale bars: 200 μm), as well as (B3) the stab needle (scale in cm). **(C)** Damage from blue light applied for 48 h from D20. The setup consisted of 2 blue LEDs, each illuminating a circular area [diameter 100 mm; scale paper (mm)]. (C1) True-color image of LED illumination, and (C2) false-color image to assess irradiance homogeneity [scale: color bar: intensity (a.u.) representing blue-light brightness]. **(D)** Different factors were applied that either are known to induce neurodegeneration (D1) and/or (D2) reactive (proliferative) gliosis in animals (see Table 1 and Supplementary Table 1). **(E)** Microglia (IB4-positive) were isolated from mouse brains, expanded in culture, and applied to MROs: They migrated into the MROs upon co-culture. Representative bright field microscopic images of microglia (E1,1’), MRO (E2,2’), and coculture of both (E3,3’). (E4) Representative images of MRO section immunostained for IB4 and DAPI. (E4’) Each circle represents one organoid (*n*). Plot depicts mean ± SD of number of microglia per MRO section at D23 (*n* = 31). Scale bars: 200 μm.

**FIGURE 2:**
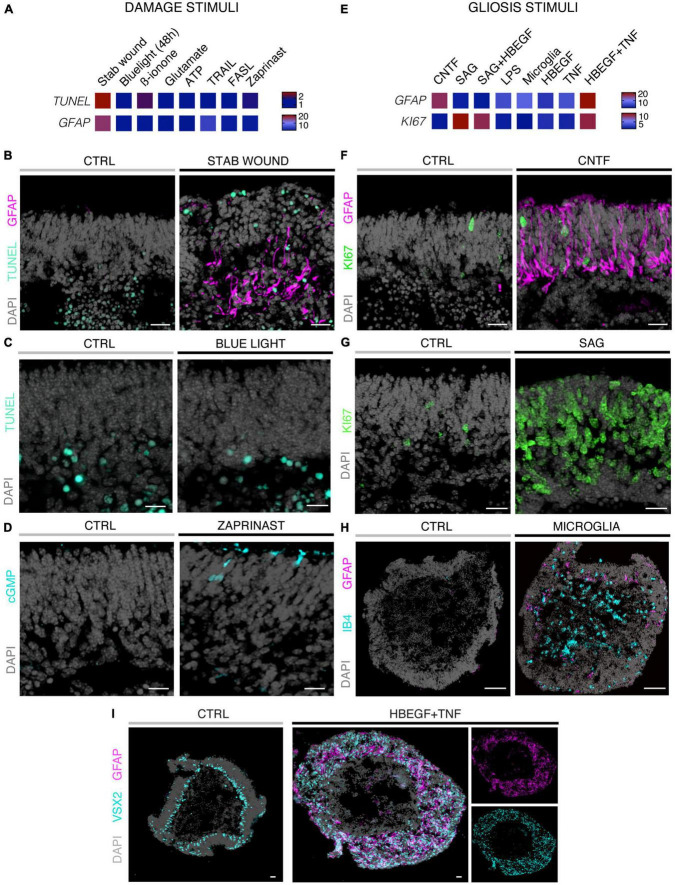
Histological analysis of MROs experimentally challenged by different treatments. **(A,E)** Heatmaps summarizing quantitative data for the two different experimental strategies indicated and explained in Figure 1 and Supplementary Table 1 (fold-change; normalized to respective control organoids; statistical analysis: Supplementary Table 2; *n* ≥ 5 organoids derived from *N* ≥ 1 independent experiment were analyzed for each challenge). Methods of experimental challenges to induce neurodegeneration and/or gliosis: All challenges were applied to 20 day-old (D20) MROs and analyzed after 5 days. **(B–I)** Representative images of immunostained MRO sections highlighting selected phenotypes using challenges and quantitative results shown in Figures 2A, E. **(B)** Stab wound caused localized increased cell death and gliosis. **(C)** Blue light treatments did not induce an increase in the TUNEL assay for cell death by D25. **(D)** Zaprinast-treated MROs showed cGMP accumulation in a subset of cells with a photoreceptor-like morphology. **(F)** Application of CNTF caused gliosis, but did not induce cell proliferation. **(G)** Smoothened agonist (SAG) induced strong cell proliferation, but no gliosis (see Supplementary Figure 1F). **(H)** Microglia (IB4-positive) migrate into the MROs upon coculture and induce gliosis. However, GFAP levels were variable within and between different MROs. **(I)** Combined HBEGF and TNF (HT) application induced a strong increase in gliosis and proliferation (not shown). Control (CTRL) MROs showed a layered structure, with MG nuclei (VSX2 +) localized in a defined layer in the inner part of the organoid epithelium. Upon HT treatment, this layered structure was lost and VSX2 + cells were distributed along the entire epithelial width. Scale bars: 20 μm **(B–G)**, 100 μm **(H–I)**.

Eight different pathology-inducing challenges were individually applied to MROs either once on day zero, or daily for up to 5 days (Figures 1A–D1) to determine if MROs are competent to reproduce a pathology and, if so, with differential phenotypes (for details see Supplementary Experimental Procedures; Table 1, Supplementary Figure 1 and Supplementary Table 1). Here, we used 20-day-old MROs since we previously reported that at this timepoint retinogenesis is complete, retinal cell composition remains stable for another 5–10 days, and the maturation stage is comparable to an early postnatal stage in mice *in vivo* (Volkner et al., 2021a). Of note, several inherited retinal diseases in patients and animals start to develop a pathology phenotype, with loss of retinal cells and subsequent deficits in visual function, during or after retinal development (Pierce, 2001; Sancho-Pelluz et al., 2008; Alves et al., 2014). To assess pathology induction, cell death and gliosis were quantified on MRO sections by TUNEL assay and by immunostaining for the hallmark marker GFAP, respectively, (Figures 2A–D, Supplementary Figures 1, 2 and Supplementary Table 2). To model physical retinal damage, we developed a standard stab-wound injury by a blunt-end needle (outer diameter: 420 μm, Figures 1B, 2A, B). TUNEL and GFAP were significantly and reproducibly induced in a localized stab wound region (Figure 2B and Supplementary Table 2). However, we detected variable (ATP, TRAIL, FASL, Zaprinast), or no (blue light, glutamate, ß-ionone) responses within the studied timeframe when using other approaches previously shown to be sufficient to induce retinal degeneration in animals *in vivo* and/or in cell culture. At commonly used concentrations, the two solvents ethanol and DMSO had no effect on their own (Supplementary Table 2). Since stab-wound injury induced both cell death and gliosis, this raised the question of whether other challenges showed no effect due to limitations in the MRO system, experimental design, or readout. Thus, we studied two in more detail: Blue light damages retinal neurons and secondarily causes gliosis *in vivo*, unless MG cells also become damaged (Chen et al., 2019). MROs were treated for 0.5, 1, 4, 6, 24, or 48 h starting on D20 with constant blue-light irradiation, and analyzed at D25 (Figures 1C, 2A, C and Supplementary Figures 2A, B). GFAP was absent in controls, but increased, albeit variably, throughout the retina after shorter and thus less damaging exposure times, but not at higher ones: This indicated that the effect was dose-dependent, and staining for activated caspase 3 (aCASP3) indicated blue light induced cell death (Supplementary Figure 2B). Thus, gliosis might be induced due to blue light-mediated damage of PRs as previously shown *in vivo* (Grimm et al., 2001; Iandiev et al., 2008). However, the damage response might still be low since 20-day-old MROs do not yet express rhodopsin at full level and lack outer segments (Volkner et al., 2021a). Further, Zaprinast, a pharmacological inhibitor of phosphodiesterase-6 (PDE6), is known to cause rod cell death via accumulation of cGMP in animal retinas, mimicking PDE6 gene mutation pathology. In control MROs, no cGMP was detected by immunostaining, and while cGMP increased in some MRO cells after 5 days of Zaprinast, TUNEL, and GFAP did not (Figures 2A, D and Supplementary Figures 1D, 2C), indicating that the inhibitory effect was just beginning or still limited. Together, our data provide evidence that the early post-mitotic MRO system used here can reproduce retinal degeneration via cell death, and respond to physical or blue-light injury with gliosis. However, in several of the challenges we did not detect any pathology in MROs, although these are known to cause pathologies in *in vivo* retinas. This raises the question of whether MROs might still be limited, due, for example, to incomplete functional maturation or a lack of certain cell components.

**TABLE 1:** Overview of the experimental challenges used for pathology modeling in organoids.

Challenge	Pathology phenotype: aim/predicted vs. detected					
	Neuronal cell death		Reactive gliosis		Cell proliferation	
Stab wound	**+**	+	**+**	+	**(+)**	nd
Blue light	**+**	−	**+**	(+)		nd
Glutamate	**+**	−	**+**	−	**+**	nd
ATP	**+**	−	**+**	−	**+**	nd
Zaprinast	**+**	−	**+**	−	−	nd
B -ionone	**+**	−		−		nd
TRAIL	**+**	−		−		nd
FasL	**+**	−		−		nd
CNTF	−	nd	**+**	+	**+**	−
HBEGF	−	nd		−	**+**	(+)
TNF	**+**	nd	**+**	(+)	**+**	(+)
HBEGF + TNF	**+**	nd	**+**	+	**+**	+
SAG		nd	**+**	−	**+**	+
SAG + HBEGF		nd	**+**	−	**+**	+
Microglia	**+**	nd	**+**	(+)	**+**	−
LPS	**+**	nd	**+**	(+)		

Potential outcomes of the pathologic challenges applied to MROs. Further details: Supplementary Experimental Procedures in Supplementary Table 1. Data summarized based on Figure 1 and Supplementary Table 1; aim/predicted (left columns) vs. detected (right columns); phenotype: +, positive; (+), low level or infrequently positive; −, not detectable; nd, not determined.

### 3.2. Modeling of differential glial and complex pathologies in MROs

We sought to further explore if, in principle, different types and levels of glial pathologic processes and a complex retinal pathology phenotype could be modeled in MROs by taking a different approach. We hypothesized that activation of defined signaling pathways by selected factors (CNTF, SAG, HBEGF, TNF, LPS) or by microglia, both previously reported to induce or mediate glial pathology alone *in vivo*, might be also sufficient in MROs (Figures 1A, E, 2E–I; details in Table 1, Supplementary Figures 1, 2 and Supplementary Table 1). Further, we tested if combined (not single) HBEGF-TNF (HT) application induces a complex phenotype in MROs, and our results presented here subsequently led to our recently published study in HROs (Volkner et al., 2022). Here, we had hypothesized this phenotype since TNF is a proinflammatory factor regulating different cell death types, and may be involved in PR degeneration (Murakami et al., 2013; Cuenca et al., 2014; Sudharsan et al., 2017; Olivares-Gonzalez et al., 2021), and both HBEGF and TNF had been shown to regulate glial pathologic processes (Bringmann et al., 2009; Inoue et al., 2013; Cuenca et al., 2014; Clarke et al., 2018; Lahne et al., 2020). Comparable to our strategy above, we assessed two parameters by immunostaining as a proxy for gliosis (GFAP) and cell proliferation (cell-cycle regulator KI67) (Supplementary Table 2). As a single factor, CNTF induced GFAP most strongly (Figures 2E, F), whereas LPS induced GFAP at lower levels, but neither induced proliferation, reproducing the induction of pathologic processes as previously reported *in vivo* (Table 1). Conversely, the SHH-signaling activator SAG, alone or in combination with HBEGF, induced massive cell proliferation (Figures 2E, G) but not GFAP (Supplementary Figures 1F, G). Application of mouse brain-derived microglia induced a low level of gliosis after 5 days of treatment (Figures 2E, H and Supplementary Table 2). This might be due to prior activation of the brain-derived microglia in culture, or their response to the MRO. Microglia, detected by immunostaining for IB4 and IBA1, reproducibly integrated with comparable numbers into MROs after only 3 days of co-culture (15.1 ± 6.6 cells per 1 mm MRO circumference; Figure 1E and Supplementary Figures 2D, E): Most were localized within the outer and inner retinal layers, and fewer on the MRO surface. Further, while HBEGF induces some cell proliferation but no GFAP, TNF induces both, although less GFAP than CTNF and less proliferation than SAG (Figure 2E and Supplementary Figure 1J). Notably, HT treatment induced the most severe pathology phenotype (Figures 2E, I): Strong GFAP expression, massive cell proliferation, and a major change in overall structure, all indicative of severe retina remodeling as previously described in animal models and patients (Jones et al., 2016a,b; Pfeiffer et al., 2020). Taken together, here we show that defined stimuli are sufficient to induce two different glial pathologic processes in MROs: Gliosis and proliferation. This provides models with differential qualities and strengths in a stimulus-dependent manner: Mild to severe, and with distinct or possibly complex pathologies. However, to establish all the presented challenges as robust pathology models will require further optimization of the experimental conditions or MRO system. Since the HT-induced phenotype in MROs presented above was the starting point for our recently published study on the HT-induced complex pathology in HROs (Volkner et al., 2022), we decided to compare the two models by studying the HT pathology in more detail in MROs, and use the results to determine if the MRO system also can be applied for mechanistic studies.

### 3.3. Combined HBEGF and TNF induces severe photoreceptor degeneration in MROs

**FIGURE 3:**
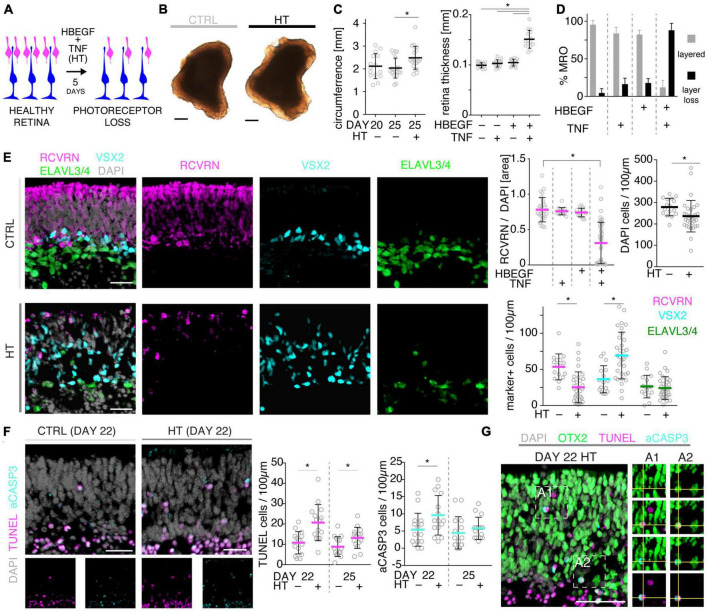
Histologic analysis of photoreceptor pathology in HBEGF-TNF (HT)-challenged MROs. **(A)** Schematic of assessing the pathologic effect of HT treatment in MROs. **(B)** Representative phase-contrast images of HT-treated and control (CTRL) MROs. **(C)** Quantification of MRO circumference and retinal epithelial thickness on images of entire central MRO sections. The apical (outer) organoid boundary was defined by DAPI, and the basal boundary by VSX2 staining. **(D)** Quantification of the change in MRO layered structure upon challenge with HBEGF (H), TNF (T), or HT in combination. *N* ≥ 3 independent experiments; *n* ≥ 10/N. **(E)** Representative images and quantitative analyses of cell-type composition of 5-day HT-treated and control MROs was assessed by immunostaining for RCVRN (PRs), VSX2 (Müller glia and bipolars), and ELAVL3/4 (amacrine and ganglion cells). **(F,G)** Representative images and quantitative analyses of cell death on HT-treated MROs and controls using TUNEL assay and immunostaining for activated caspase 3 (aCASP3). **(G)** Immunostaining for OTX2, marker of photoreceptors and bipolar cells, eco labels with TUNEL- and aCASP3-positive cells. Circles represent individual organoids (n) based on *N* ≥ 3 independent experiments (*n* ≥ 5/N). Error bars: SD. *Statistically significant (see Supplementary Table 3). Scale bars: 200 μm **(B)**, 25 μm **(E–G)**.

To further explore the competence of this MRO system to model pathologies and to study underlying mechanisms, we sought to establish and characterize the HT-induced pathology in MROs, termed the HT-MRO model, in more detail: The phenotype was one of the more prominent ones among all challenges tested here, and we wondered if it not only involves glial pathologies but also PR degeneration (Figure 3A), and thus a complex phenotype like in HROs (Volkner et al., 2022). Using phase-contrast microscopy, we observed that the optical properties of living MROs visibly changed, and the retinal epithelial thickness (the bright area in Figure 3B) did not decrease within 5 days of HT treatment. Quantitative analysis of immunostained HT-MRO cross-sections confirmed this (Figure 3C and Supplementary Table 3): The average circumference increased 1.22-fold (*p* = 0.0051, *N* = 4 independent experiments with *n* = 20/N MROs) and retinal thickness 1.5-fold (*p* < 0.001, *N* = 3 independent experiments with *n* = 15/N MROs) compared to controls. Further, combined (not separate) HT treatment induced a severe loss in retinal layered structure and cells (Figures 3D, E). In control MROs, immunostaining for cell-specific markers showed an ordered retinal structure: PRs (RCVRN) in the outer retina, amacrine, horizontal, and retinal ganglion cells (ELAVL3/4), as well as MG (VSX2) in the inner retina. In contrast, all these cell types become displaced in HT-MROs, and quantitative analysis indicated a significant 60% loss of PRs (*p* < 0.0001, *N* = 3, *n* ≥ 9/N), but not upon HBEGF or TNF treatment alone (Figure 3E and Supplementary Figure 3A). To determine if cones, which represent a low percentage of all PRs (RCVRN labels rods and cones), might also be affected we assessed the cone marker OPN1SW, which was detectable in controls but not in HT-MROs (Supplementary Figure 3B). Interestingly, although a large number of PRs were lost, HT treatment reduced the total cell number based on cell nuclei counts by only 15% compared to controls (*p* = 0.0373; Figure 3E) raising the question of whether other cells might increase in number. Analysis of ELAVL3/4, a marker of amacrine, horizontal, and retinal ganglion cells, showed no changes in cell numbers (Figure 3E). However, VSX2 cells, which mostly represent Müller glia, but also a subpopulation of bipolar cells, increased 1.92-fold (Figure 3E, *p* = 0.0005, *N* = 3, *n* ≥ 5/N). TUNEL assay and immunostaining for aCASP3 both support increased cell death within the outer retina of HT-MROs, confirming retinal degeneration (Figures 3F, G and Supplementary Figure 3C): After 2 and 5 days of HT treatment both markers show more positive cells than controls—most strongly at D22 (TUNEL *p* = 0.0011; aCASP3 *p* = 0.0387; *N* = 3, *n* = 15 per variable). Cell death marker positive cells overlap with OTX2, which label all PR cell nuclei in the outer retina and a subpopulation of bipolar cells in the inner retina of MROs (Figure 3G) as previously reported in animals and MROs (Volkner et al., 2016, 2021a). Together, our data demonstrate that application of HT for 5 days is sufficient to induce not just gliosis but also severe PR degeneration. Since aCASP3 indicated an apoptosis-mediated PR cell death, we also explored the expression of cyclic guanosine monophosphate (cGMP), which has been linked to PR degeneration in many genetic retinal diseases (Tolone et al., 2022). After 5 days of HT treatment, cGMP was slightly and variably increased in some but not the majority of MROs (Supplementary Figures 3D, E). Since cGMP might be related to apoptotic and non-apoptotic cell death, and one might be induced by the other or both could occur in parallel, we studied receptor-interacting serine/threonine-protein kinase 3 (RIPK3) and poly (ADP-ribose) (PAR) (Supplementary Figure 3F): These regulate non-apoptotic cell death, and DNA damage and inflammation, respectively, in different forms of retinal diseases, and may be induced by TNF (Murakami et al., 2011, 2013; Humphries et al., 2015; Tao et al., 2022). Here, we observed that neither regulator increased upon 2 or 5 days of HT treatment. Taken together, our data indicate HT-induced PR degeneration, possibly via apoptosis, and although PRs become severely depleted, the retina becomes thicker rather than thinner, suggesting that the lost PRs might be replaced by other cells, likely MG, indicative of a complex retinal pathology.

### 3.4. HBEGF-TNF treatment induces a complex retina pathology in MROs

**FIGURE 4:**
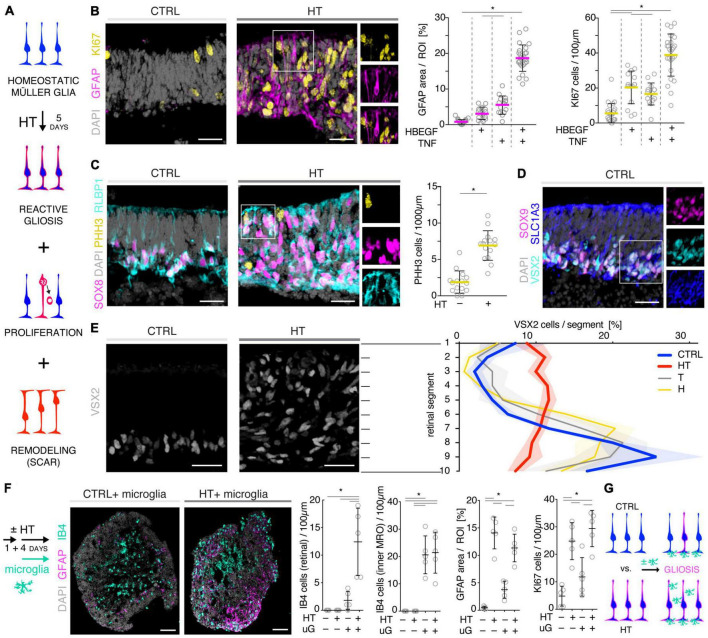
Investigation of Müller glia and microglia in HBEGF-TNF (HT)-challenged MROs. **(A)** Schematic of monitoring glial pathologies in HT-treated MROs. **(B,C)** Representative images and quantification of GFAP (gliosis marker), Ki67 (proliferation) and PHH3 (mitosis marker). **(C)** PHH3 co-localizes with the Müller glia (MG) markers SOX8 and RLBP1. **(D)** Co-localization with the glial markers SOX9 and SLC1A3 confirmed expression of VSX2 in MG in MROs. **(E)** Retina remodeling was quantified by assessing the displacement of MG nuclei (VSX2 +) along the radial axis. The position of each VSX2 + nucleus was measured, and the relative distribution quantified. In control MROs, VSX2 + nuclei localized to a layer in the inner retina. After HT treatment, this layering was lost and VSX2 + nuclei were spread evenly along the entire width of the retina. **(F)** Schematic of microglia and HT-MRO coculture. Representative images and quantitative analysis of MRO sections stained for selected markers: GFAP (reactive gliosis), IB4 (microglia), Ki67 (proliferation), and DAPI (cell nuclei). **(G)** Schematic of results shown in **(F)**. Circles represent individual organoids (*n*) based on *N* ≥ 3 independent experiments, *n* ≥ 5/N **(A–E)** and *N* = 1, *n* ≥ 4 **(F)**. Error bars represent SD. *Statistically significant (see Supplementary Table 3). Scale bars: 25 μm.

**FIGURE 5:**
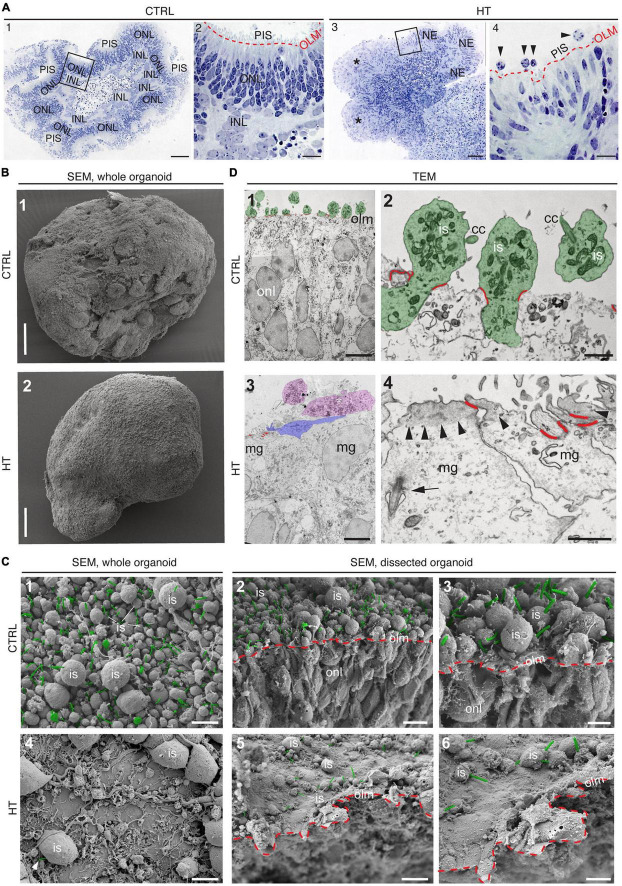
(Ultra)structural studies of the HBEGF-TNF (HT) model in MROs. **(A)** Histology of control (CTRL) (A1,A2) and HT-treated (A3,A4) MROs. (A2,A4) Red dashed lines: Outer limiting membrane (olm); photoreceptor inner segments (PIS); squares in (A1,A3) indicate the regions shown in (A2,A4), respectively. Some areas of HT-treated organoids show an ordered array of neuroepithelium (NE), while other areas are in complete disarray [*in (A3)], and show some potential ectopic cells [arrowheads, (A4)]. Scanning [SEM, **(B,C)**] and transmission [TEM, **(D)**] electron microscopy analysis of CTRL and HT-treated MROs. (B1) Whole-organoid SEM: Surface of CTRL MROs densely packed with photoreceptor inner segments (is). (C1) MRO surface shown at higher magnification. Connecting cilia are pseudocolored in green. (C2) SEM of a dissected organoid. Red dashed line: OLM; green: Connecting cilia. (C3) Region of C2 shown at higher magnification. (B2,C4–C6) In contrast, HT-MROs have very few PISs on the surface. (B2) Whole organoid, (C4) surface at higher magnification, only one connecting cilium (green) visible in this field of view. (C5) SEM of a dissected HT-treated organoid. Red dashed line: OLM; only a few PISs with connecting cilia (green). (C6) Region of C5 at higher magnification. (D1,D2) TEM of a CTRL organoid. (D1) Overview: Inner segments are pseudocolored in green, the junctions of the OLM in red. (D2) Higher magnification showing several photoreceptors (pseudocolored in green), inner segments (is), connecting cilia (cc), and junctions of the OLM (red). (D3,D4) TEM of an HT-treated MRO. (D3) Overview: Some potential inner segments or ectopic cells are pseudocolored in magenta, one apically extended Müller glia cell process (mg) is pseudocolored in blue, the junctions of the OLM in red. (D4) Some apical cell surfaces are shown (Müller glia, MG). Note the accumulation of electron-dense material (probably actin) in the apical cortex (arrowheads), the arrow points to a pocketed primary cilium, OLM junctions pseudocolored in red. ONL, Outer nuclear layer; INL, Inner nuclear layer. Scale bars: 100 μm (A1,A3,B1,B2), 20 μm (A2,A4), 5 μm (C1,C2,C4,C5,D1,D3), 2 μm (C3,C6), 1 μm (D2,D4).

Given our data above, we hypothesized that the HT-MRO model might reproduce a complex phenotype with combined PR and MG pathologies (Figure 4A). We quantitatively compared MROs treated with HBEGF, TNF, or combined HT (Supplementary Table 3): GFAP is more strongly upregulated after HT treatment than single factors (Figure 4B; *n* = 5/N, *N* ≥ 3 per variable). Counts of the cell-cycle marker KI67 showed that cell proliferation was higher after HT treatment than single factors or control (Figure 4B and Supplementary Figure 1J). To determine if proliferating cells are MG, and if they undergo cell division, we costained with the MG marker SOX8 and the mitosis marker PHH3 (Figure 4C). SOX8 stains MG nuclei, as confirmed by costaining with the glia marker RLBP1, indicating a cytoplasmic labeling throughout the radial spanning MG cell, confirming previous reports (Loffler et al., 2015; Volkner et al., 2021a). Upon HT treatment, there were more PHH3-positive cells than in controls (Figure 4C), supporting MG proliferation. To quantitatively assess the qualitative pathologic retinal dyslamination, as observed in complex pathologies and in remodeling (Jones et al., 2016a,b; Pfeiffer et al., 2020; Volkner et al., 2022), we measured radial displacement of cell nuclei across the retinal epithelium (Figures 4D, E and Supplementary Figure 3A): In controls, VSX2 labels MG and some bipolar cell nuclei (Figure 4D), both localized in a narrow band within the retinal inner (INL) but not outer (ONL) nuclear layers—comparable to animal models (Loffler et al., 2015; Volkner et al., 2021a). In HT-MROs (not each factor alone), VSX2 nuclei became evenly redistributed throughout the entire retinal epithelial width. Histological studies of MRO cross-sections at the (ultra)structural level by light microscopy (Figure 5A), MRO wholemounts by scanning electron microscopy (Figures 5B, C), and MRO cross-sections by transmission electron microscopy (Figure 5D), further confirmed our hypothesis: As previously established (Volkner et al., 2016, 2021a), the ONL and INL can be discriminated based on nuclear position, structure, and staining in control MROs (Figures 5A1, A2). PRs showed characteristic morphologies and an ordered organization, with PR inner segment-like structures (PISs) located outside of the apical retinal border, which sometimes showed a connecting cilium and potential nascent outer segment (Figures 5A1, A2, B1, B2, C1–C3, D1, D2). The outer limiting membrane (OLM) is known to be formed by cell junctions between PRs and MG, and imaging shows PRs with mitochondria-rich PISs alternating with other cells, most likely MG (Figure 5D2). PIS loss is a clinical biomarker of PR degeneration and irreversible vision loss (Cuenca et al., 2014; Litts et al., 2015; Li et al., 2018). Notably, most apical structures formed by PISs and the OLM are lost in HT-MROs (Figures 5A3, A4, B2, C4, C5, D3, D4). This can also be seen on en-face SEM images of MROs: Controls show a high density of PRs based on many visible PISs (Figures 5C1–C3), and these are lost In HT-MROs, resulting in a rather smooth retinal surface structure (Figures 5C4–C6). Related to the pathologic changes of PRs described above, we also observed that the ONL is frequently replenished or overgrown by a disordered cell mass with more cytoplasmic cell area between cell nuclei compared to controls (Figures 5A1–A4). These observations are in line with the above-described glial proliferation and gliosis in the HT-MRO model. Ultrastructural images show not only that the most apical ordered structure of the OLM is lost in pathologic regions (Figures 5C3–C5, D3, D4), but also that the contiguous region below the OLM is affected: The apical cell layer is mostly formed by cell contacts between amorphous cells larger than in controls (Figures 5A1–A4, D1–D4), some of which infrequently show a pathologic perpendicular orientation. These data support the hypothesis that lost PRs become replaced by MG, some MG overgrow remaining PR cells or replace lost ones (Figure 5D3, blue pseudocolored cell), as reported in animal models and patients (Guidry et al., 2002; Sullivan et al., 2003; Wu et al., 2003; Lewis et al., 2010; Jones et al., 2016b; Zanzottera et al., 2016; Edwards et al., 2017; Li et al., 2018, 2021), which might be part of a process forming glial seal-like scars. Notably, some cells are localized ectopic of the OLM (Figures 5A4, D3), suggesting that PRs might degenerate via cell extrusion as we previously also observed in HT-HROs (Volkner et al., 2022). To determine if the complex phenotype, including the increased retinal thickness, is transient or remains even upon removal of HT, we applied HT to 20-day-old MROs for 5 days daily, and analyzed them at D25 and D31 (Supplementary Figure 4). We observed that the increase in retinal thickness and reactive gliosis still remained beyond HT application, although there was no more pathologic cell proliferation. Our data indicated a complex pathological process that might be reproducing processes relevant to complex pathologies, which have previously been associated with inflammation. Thus, we wondered if microglia might be attracted to the retina more strongly or differently compared to controls and, if so, whether these particularly localized to the outer (retinal epithelium) or inner region within MROs. Further, microglia have been shown to regulate and interact with Müller glia and reactive gliosis. To explore this, we applied HT for 5 days, and added microglia to them as a co-culture after one day of HT treatment (Figure 4F and Supplementary Figure 5). Immunostaining analysis and quantification of reactive gliosis using GFAP staining as a proxy showed that microglia and HT treatment induce lower and higher levels of reactive gliosis, respectively, compared to untreated controls, confirming earlier results (Figure 1), and gliosis upon treatment with microglia combined with HT was comparable to HT alone (Figure 4F). Interestingly, microglia invaded the inner part of MROs in comparable numbers when applied with or without HT: However, microglia in HT-MROs localized more numerously to the retinal epithelial layer than in controls (Figure 4F). Together, our data suggest that microglia become attracted to degenerating PRs or the apical processes of MG undergoing gliosis (Figure 4G). MROs treated with or without HT but with no microglial co-culture showed no microglia based on IB4 staining (Supplementary Figure 5). Further, we wondered if the HT-induced pathology affects only post-mitotic retina or also immature neurons in the developing retina. To explore this, we applied HT to MROs from D15 to D18 during retinogenesis (Supplementary Figure 6, *n* = 5/N, *N* = 1 per variable), which represents the timeframe when the majority of retinal cells are still being generated (Volkner et al., 2016). Further, application of DAPT, an inhibitor of the Notch signaling pathway, during the same timeframe served as a positive control, which is well-known to induce premature differentiation (Volkner et al., 2016). We performed immunostaining for CRX, ASCL1, TUNEL, EdU, and GFAP to monitor changes in PRs, retinal progenitors, cell death, proliferation, and gliosis, respectively. HT treatment did not show any effect on these parameters, whereas Notch inhibition led to the expected increase in PRs, a related decrease in proliferation and a depletion of retinal progenitors. Interestingly, DAPT caused an increase in ectopic PRs, whereas HT did not cause any pathologic change of the parameters analyzed. Thus, the DAPT effect might be explained by the forced cell overproduction, resulting in overcrowding-induced cell extrusion, which has been observed during embryonic development (Volkner et al., 2016). Together, our data demonstrate that HT application is sufficient to induce a severe neuropathology in post-mitotic but not developing MROs: PR degeneration develops, together with MG gliosis, proliferation, and scar formation within 5 days. Potentially, other features of retinal remodeling which result in retinal dyslamination and thickening also occur: taken together, these are characteristics of complex pathologies in animals and patients (Sarks et al., 1988; Guidry et al., 2002; Sullivan et al., 2003; Wu et al., 2003; Jacobson et al., 2006; Aleman et al., 2007; Pow and Sullivan, 2007; Lewis et al., 2010; Alves et al., 2014; Bird et al., 2014; Jones et al., 2016b; Zanzottera et al., 2016; Edwards et al., 2017; Dolz-Marco et al., 2018; Li et al., 2018, 2021; Sadda et al., 2018; Chen L. et al., 2020; Guymer et al., 2020; Pfau et al., 2020; Zouache et al., 2020). In conclusion, MROs are competent to effectively reproduce distinct pathologic processes and complex phenotypes.

### 3.5. Pharmacologicals differentially prevent photoreceptor and glial pathology

**FIGURE 6:**
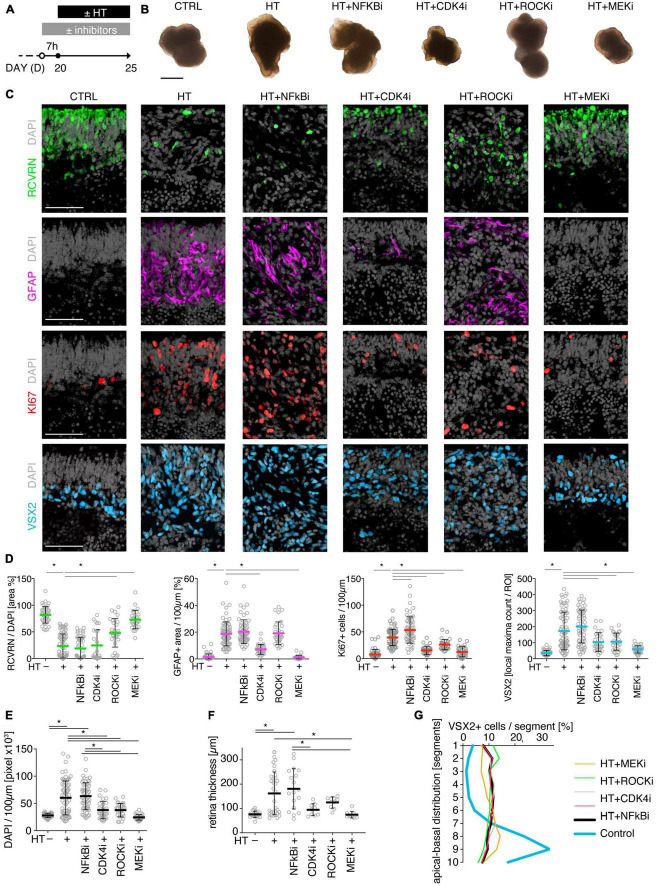
Pharmacological-based intervention of the HT-induced pathologies. **(A)** Experimental design. MROs were differentiated until D20 and then treated with HBEGF-TNF (HT). Specific inhibitors of NFkB, ROCK, MEK, and CDK4 were applied starting 7 h before HT addition and then throughout the HT treatment period together with HT. MROs were analyzed at D25. **(B)** Representative phase-contrast images of MROs in culture on D25. **(C)** Representative images and **(D,E)** quantitative analyses of CTRL, HT, and HT + pharmacological MRO sections immunostained for RCVRN (photoreceptors), GFAP (reactive glia), KI67 (cell proliferation), VSX2 (Müller glia, and some bipolars) and DAPI (nuclei). **(F)** Retinal epithelial thickness was determined on microscopic images of entire central MRO sections. The apical (outer) organoid boundary was defined by DAPI, and the basal boundary by VSX2 staining. **(G)** Retina remodeling was quantified by assessing the displacement of MG nuclei (VSX2 +) along the radial axis. The position of each VSX2 + nucleus was measured, and the relative distribution was quantified. Each circle represents one MRO (*n*) based on *N* = 3 independent experiments (*n* ≥ 10/N). Error bars represent SD. *Statistically significant (see Supplementary Table 4). Scale bars: 500 μm (B), 50 μm **(C)**.

**FIGURE 7:**
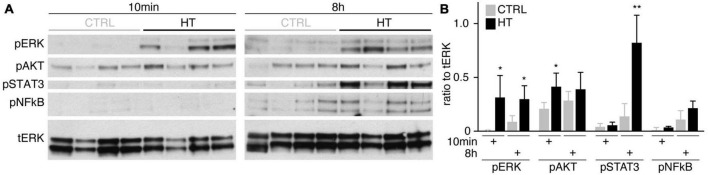
Signaling pathway activation in the HT-induced pathology model. **(A)** Western blot analysis of whole MRO lysates collected after 10 min and 8 h of HT treatment compared to solvent controls (*n* = 4 MRO per variable) to determine phosphorylation of ERK1/2 (pERK), AKT (pAKT), STAT3 (pSTAT3), and NFkB (pNFkB). **(B)** Immunoreactive bands were quantified, and all signals were normalized to total ERK (tERK); ratios were determined for individual MROs. Supplementary Figure 7 shows related data. Each circle represents one individual MRO (*n* = 4) based on *N* = 1 independent experiments. Error bars represent SD. Statistically significant: *p < 0.05, **p < 0.01.

To determine if MRO models facilitate studies of PR and glial pathomechanisms, we sought to explore this in the HT-MRO model (Figure 6). We applied pharmacological inhibitors for signaling pathways previously reported to be involved in the HT-HRO model or in other animal pathology models (details on pharmacological are listed in Supplementary Experimental Procedures). In brief, previous studies have shown that MEKi (UO126; Duncia et al., 1998; Favata et al., 1998) is a chemical dual inhibitor for mitogen-activated protein kinase 1 and 2 (MEK1/MEK2) of the mitogen-activated protein kinase (MAPK), also known as extracellular signal-regulated kinase (ERK) signaling pathway, which controls PR cell survival and gliosis (Kinkl et al., 2001; Swain et al., 2007; Donovan et al., 2011; Besirli et al., 2012; Sardar Pasha et al., 2017; Dong et al., 2022; Zeng et al., 2022). Notably, MEKi completely prevented pathogenesis in the HT-HRO model (Volkner et al., 2022). NFkBi, a nuclear factor-kappa B (NFkB) signaling inhibitor, modulates PR neuroprotection and MG proliferation (Rana et al., 2014; Wang et al., 2017; Palazzo et al., 2023). ROCKi, a rho-associated kinase (ROCK) inhibitor, may reduce MG proliferation and gliosis (Tura et al., 2009; Zhang et al., 2015; Halasz et al., 2021). CDK4i blocks cyclin-dependent kinase 4 (CDK4), and drives cell proliferation and possibly cell death (Arsenijevic, 2016). Inhibitors were applied 7 h before HT, and subsequently added daily together with HT until D25 (Figure 6A, *N* = 3 independent experiments, *n* ≥ 10 MROs/N and variable). As described above, phase-contrast live microscopy of HT-MROs (Figure 6B) showed an increased retinal epithelium width and impairment of its even surface, which was reduced at least to some extent by CDK4i, MEKi, and ROCKi, but not NFkBi. We quantitatively assessed PR loss, MG cell number, proliferation and gliosis using our established histological analysis (Figures 6C, D and Supplementary Table 4): HT caused a two-thirds decrease in PRs (RCVRN, *p* < 0.0001), MG proliferation (VSX2 and Ki67, both *p* < 0.0001) and gliosis (GFAP, *p* < 0.0001). Next, we compared the effect of the pharmacological on HT-MROs (Figures 6C, D and Supplementary Table 4): Notably, MEKi affected the development of all assessed pathologic changes (each *p* < 0.0001), completely preventing PR loss, as well as MG proliferation and gliosis. Qualitatively, even the overall impairment in retinal layering was reduced, based on PRs and DAPI. In contrast, ROCKi did not prevent gliosis, but reduced the loss of PRs by about half (*p* < 0.0001) and prevented MG proliferation (*p* < 0.0001). NFkBi showed no effect. We applied CDK4 to determine if a general block of the cell cycle machinery protected the MROs from PR degeneration, but it did not, although proliferation was reduced. Further, CDK4i reduced gliosis by about half (*p* < 0.0001). Quantitative analysis also showed that MEKi, CDK4i and ROCKi prevented an increase in total cell number and retinal thickness (Figures 6E, F), while only MEKi showed a slight effect on radial displacement of MG cell nuclei (VSX2) across the retinal epithelium (Figure 6G). Together, MEKi was most powerful in preventing the complex phenotype with PR and glial pathologies. ROCKi, but not NFkBi or CDK4i, rescued some PRs, whereas CDK4i but not NFkBi or ROCKi reduced gliosis, while MEKi, ROCKi, and CDK4i reduced MG proliferation. To confirm that the HT-induced pathology involves regulated pathomechanisms, we performed western blot analysis to assess the activity of the major signaling pathways ERK1/2, AKT, NFKB, and STAT3, which previously had been associated with various types of retinal degeneration (Kassen et al., 2007; Donovan et al., 2011; Xia et al., 2011; Rhee et al., 2013; Jiang et al., 2014; Rana et al., 2014; Wang et al., 2017; Zeng et al., 2022; Palazzo et al., 2023). We analyzed MROs at 10 min and 8 h after HT treatment, and observed an increase in phosphorylation of ERK (10 min and 8 h), AKT (8 h), and STAT3 (8 h), but not in NFkB, indicating differential increased activation (Figure 7 and Supplementary Figure 7). In conclusion, our results reveal that HT treatment is sufficient to induce a complex pathology in MROs with differentially regulated PR and glial processes indicating pathomechanistic access to MROs.

## 4. Discussion

**FIGURE 8:**
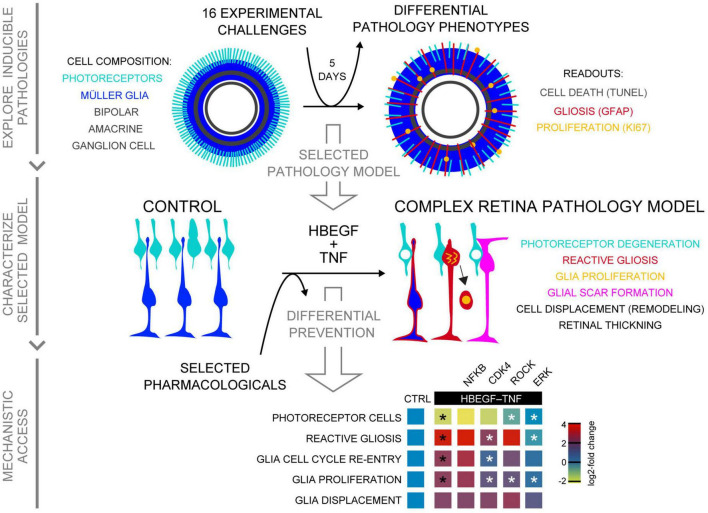
Summary of induced retina pathology modeling in the MRO system. Schematic shows, from top to bottom, summary of all three phases of this study: Exploration of inducible neuropathology in MROs; characterization of HT-induced MRO pathology; and mechanistic study with pharmacological. Summary heatmap of pharmacological study; heatmap scale shows log2-fold changes compared to CTRL (based on data in Figures 6D, G). Statistically significant as indicated in Figures 6D, G: black asterisks compared to CTRL, white asterisks compared to HBEGF–TNF.

Future personalized preventive and regenerative therapeutic treatments for a broad spectrum of patients with neurodegenerative diseases might be most effective if treatments are defined by the individual patient’s pathology stage and phenotype. Thus, effective inducible pathology models with a defined pathologic process and progressive phenotypes will facilitate preclinical therapy development, translation, and clinical optimization. Human (HRO) and mouse (MRO) retina organoids offer complementary advantages to facilitate studies in animal models and patients, but organoid technology is still in the pioneering phase, and many questions remain. For example, it is still unclear to what extend the current systems are sufficient to reproduce all stages and processes of pathologies, and there have been no longer-term studies exploring this in detail. Here, we demonstrate experimentally inducible pathology models with differential phenotypes in MROs upon application of challenges known to be sufficient to induce a neuropathology in animal *in vivo* models (Figure 8 and Table 1). Interestingly, our data indicate that although MROs are a more reduced system than the retina *in vivo*, they are still sufficient to reproduce major pathologic processes as previously observed *in vivo* upon different types of challenges, and are potentially relevant for a wide variety of diseases in patients. However, some treatments showed no effect, and thus our data might provide an insight into retinal vulnerability and organoid system limitations, and thus starting points for further optimizing the MRO system. Notably, the development of MRO pathology models presented here was the basis for our recently reported human retinal pathology model induced by treatment with combined HBEGF-TNF (HT), which revealed a pathomechanism of PR degeneration via cell extrusion. Here, we further characterized the MRO model and show that application of HT, two factors previously associated with neuropathologies in animals and patients *in vivo* (Bringmann et al., 2009; Inoue et al., 2013; Murakami et al., 2013; Cuenca et al., 2014; Sudharsan et al., 2017; Clarke et al., 2018; Lahne et al., 2020; Olivares-Gonzalez et al., 2021), is sufficient not only in HROs but also in MROs to induce a complex pathology: PR degeneration, gliosis, glial proliferation, scar formation, and retinal dyslamination. Further, pharmacological inhibitors for selected signaling pathways completely prevent (MAPK) or differentially affected (RHO/ROCK, NFkB, CDK4) pathologic changes in PRs and MG, indicating that there is mechanistic access in MROs. Interestingly, our data show that the major pathological processes induced by HT in MROs are comparable to our previous observations in HROs (Supplementary Table 3B), although the underlying mechanisms of PR degeneration seem to differ. While PRs degenerate via live cell extrusion and subsequent cell death in HROs, in MROs they undergo cell death in the retina *in situ* and some PRs appear ectopically—indicative of cell extrusion (Volkner et al., 2022). Thus, PRs in MROs might degenerate upon HT by two different mechanisms at the same time, or undergo cell death-induced extrusion (Gudipaty et al., 2018). Alternatively, HT might induce the same complex pathology in MROs and HROs, but MROs (Volkner et al., 2021a), and not HROs (Volkner et al., 2022), develop in addition the previously observed spontaneous PR degeneration prematurely. In conclusion, this study shows that MROs can reproduce different pathology phenotypes and processes, providing mechanistic access and a useful primary system to develop pathology models prior to their transfer to HROs and animals *in vivo*, and then their validation in patients.

Many different established, inducible animal models are continuously advancing neuropathology research (Supplementary Table 1). Here, we started to establish several in MROs. Some show different phenotypes: Stab wound, blue light, SAG, CNTF, and HT; but others do not, which provides an insight into the competence and limitations of the MRO system. Stab-wound injury reproducibly causes localized PR degeneration and gliosis, with neighboring regions remaining unharmed, whereas pathology-inducing stimuli reaching all cells within an organoid, like extrinsically applied cytokines, effectively induce a pathologic response throughout the MRO. Depending on the stimulus, we observed different pathology qualities and magnitudes, like high (CNTF) or low (microglia, LPS, blue light) gliosis levels, cell proliferation (SAG), or PR degeneration and gliosis in combination (HT). The CNTF model is well-characterized in mice *in vivo*, and thus could readily be used for comparative studies between MROs, HROs, and mice. SAG induced the strongest proliferation response in post-mitotic retinal models. We, and other researchers, have observed an age-dependent restriction of MG proliferation in mammals and other species (Fischer, 2005; Loffler et al., 2015), whereas in zebrafish MG more readily proliferate upon injury or experimental stimulation (Goldman, 2014; Lenkowski and Raymond, 2014). Our SAG data suggest that glia, and possibly even neurons, re-enter or activate parts of the cell cycle, which may contribute to neurodegeneration (Robel et al., 2011; Zencak et al., 2013; Arsenijevic, 2016; Sardar Pasha et al., 2017). This SAG model might also facilitate studies on the acquisition and maintenance of cell homeostasis, and glia-derived neuronal regeneration (Loffler et al., 2015; Todd and Fischer, 2015; Sardar Pasha et al., 2017; Lahne et al., 2020). Interestingly, for some challenges we did not detect a pathologic change, which might be due to experimental design, readouts, or MRO limitations. Notably, blue light induced gliosis, but little cell death; unlike *in vivo*, the overall degeneration was rather low and not progressive (Geiger et al., 2015). Possibly, blue light, ATP, and TRAIL each induce very little cell death in some but not all organoids, which might reflect MRO heterogeneity or other deficits. For example, longer-term treatments might be necessary to develop a full response. Alternatively, for some challenges 20-day-old MROs might not yet be mature enough, and thus functional enough, to develop a pathology, e.g., to reach the cGMP (zaprinast) or opsin (beta ionone) levels required for inducing cell death (Bowes et al., 1990; Volkner et al., 2021a). Further, other cell components not yet included in MROs, e.g., vasculature, microglia, or RPE, might be required for some pathologies. It is still not known to what extend these and other cells are needed in the retina to complete maturation or maintain homeostasis. Interestingly, we show that merely co-culturing control MROs with microglia results in their invasion, whereas more numerous microglia invade the outer retina in pathologic MROs, possibly due to reactive MG and ongoing PR degeneration. Introducing defined additional cell types, possibly even with different activation states or other manipulation, at any point of an experiment generally offers a useful strategy to determine their contribution to the dynamics of pathogenesis. Our data raise the question of whether microglia induce gliosis due to pre-activation, physical invasion, cell contact, response to the MRO environment, or other factors. Further, which signals and cell types attract microglia upon different types and mechanisms of neuropathology? In conclusion, several distinct pathologic processes can be induced and reproduced in MROs, providing experimental phenotypic models. MROs are a reduced system, which can be seen as a limitation, since not all pathologies are readily reproduced. However, organoid reductionism is also advantageous: It focuses on major cell types, and others can be introduced instead of ablated *in vivo*, a process which may even cause pathologies (Zhang et al., 2018). Thus, MROs provide an assay system to determine if experimental challenges are sufficient to induce a pathology, and to develop defined and complex phenotypic models by stimulating (combinations of) specific pathologic processes. Interestingly, inherited retinal degenerative diseases may present with embryonic, postnatal, juvenile, and adult onset, and these onsets might depend on different vulnerabilities related to retinal maturation, age, or other age-related environmental factors. Thus, future follow-up experiments will be aimed at determining whether different organoid cell compositions, maturation states, and species have different vulnerabilities and pathomechanisms upon different challenges. Our data suggest that organoid systems could facilitate this work since they are, theoretically, available in unlimited numbers, and MROs are generated faster than HROs. Organoids are a reduced retinal model system (e.g., without microglia, vasculature, RPE), which provides advantages for larger-scale experiments and for studying the function of defined cell components by adding them into organoids. We show that microglia differentially localize in MROs with and without pathology. Culture systems with primary animal- and human-derived retinas offer a complete retinal system, but are limited due to low patient donor numbers and ethical considerations. Further, primary retinas readily and spontaneously develop reactive gliosis and degenerate upon culture (Loffler et al., 2015; Sardar Pasha et al., 2017; Volkner et al., 2021a) due to tissue dissection and cell-culture stress, whereas MROs only degenerate when cultured for longer (Volkner et al., 2021a), and HROs may last for at least 260 days (Volkner et al., 2022). Further, we and other researchers have shown that some HRO systems provide a cone-rich retina with some of the features found in the human macula, whereas most animal models lack both. Still, organoid system technologies need to be further optimized to support complete functional maturation and longer-term maintenance of homeostasis at low variance.

The complex HT-MRO pathology model established here shows that HT treatment is not just synergistic and sufficient in the human model but also in the mouse model, suggesting that this might also apply to animals and possibly patients *in vivo*. An HT-induced *in vivo* model in conjunction with HT-MROs, HT-HROs, and data from patients with complex pathologies might facilitate comparative and synergistic mechanistic studies of several still incompletely understood processes which are key for many neurodegenerative pathologies—specifically advanced AMD, complex IRDs/MDDs, and (secondary) degeneration in most others. For example, it will be interesting to determine if pathogenesis is comparable in all three systems: specifically, if PRs degenerate via cell extrusion in mice in the same way as in HT-HRO, which we hypothesized might reproduce a pathology in AMD and other diseases (Volkner et al., 2022). Further, it has not yet been possible to effectively induce and study extensive pathological glial proliferation and scar formation, which might have beneficial or detrimental functions in animal models. Thus, the HT model might reproduce features of retina remodeling in advanced AMD (Fritsche et al., 2014; Sadda et al., 2018; Pfeiffer et al., 2020; Fleckenstein et al., 2021), and at late stages of most other retinal pathologies (Milam et al., 1998; Sudharsan et al., 2017) which have not yet been modeled in animals. Further, HBEGF and TNF are each sufficient to induce MG-derived proliferation and thereby neuronal regeneration in zebrafish (Lahne et al., 2020), raising the question of whether some pathologies in mammals are misregulated regeneration responses. Together, it is still unclear to which extent HT induces comparable pathologic processes in animals and human models which differ at various levels: Genome, cell composition (rod- vs. cone-dominant), and *ex*- vs. *in vivo* environments. Although several studies have shown that TNF/EGF signaling may contribute to many neuropathologies throughout the nervous system and retina (Inoue et al., 2013; Murakami et al., 2013; Sudharsan et al., 2017; Olivares-Gonzalez et al., 2021), it is still unclear to what extent HT treatment might contribute to diseases.

We previously developed the MRO system utilized here to facilitate experimental studies, and the presented data further support this. We reasoned that if MROs at the intra- and inter organoid level are very different under control conditions in terms of cell composition, maturation, or phenotype stability (e.g., some undergo cell stress and others not), this might amplify the variances of experimental changes thereby limiting their detection and quantitative analysis. If so, a pathology model might show highly variable phenotypes within and between organoids. However, we did not observe this for the HT model, supporting the robustness of this MRO system. Pathologic changes developed across the majority of MROs from the same and from different batches, and across the retina within each MRO. Spontaneous degeneration under control conditions is one remaining limitation for long-term MRO experiments. So far, organoid studies have focused on development and neonatal stages, and a short timeframe after the end of retinogenesis. There have only been two longer-term studies (Chen et al., 2020; Volkner et al., 2021a) ranging between 21 and 60 days. Several reports show that organoids, even in different systems, show increased cell death starting at D35 (Eiraku et al., 2011; Hiler et al., 2015; Chen et al., 2016; Volkner et al., 2016, 2021a; Ito et al., 2017; DiStefano et al., 2018; Ueda et al., 2018; Brooks et al., 2019; Decembrini et al., 2020). Only two studies so far have explored pathologies using different MRO protocols: Mookherjee et al. (2018) reproduced and rescued a cilia phenotype in D32 MROs. Ito et al. (2017) established toxin-induced PR cell death by interfering with PR gene expression in D23 MROs treated for 4 days with inverse ERRβ agonists. However, analysis was limited by ongoing degeneration under control conditions. We confirmed here that our MRO system shows no additional spontaneously cell death or gliosis for about 5–10 days after completing retinogenesis (Volkner et al., 2021a). Notably, in early post-mitotic *in vivo* retinas and MROs, there is still some developmental cell death but this does not inflict gliosis (Volkner et al., 2021a), whereas animal IRD models start pathogenesis with gliosis at this age (Sancho-Pelluz et al., 2008). Here, we induced gliosis at this stage in MROs, indicating competence of MG to undergo a pathologic response similar to the *in vivo* response. We also tested challenges known to be insufficient to induce a pathology *in vivo* without prior additional retinal damage, for example, HBEGF-induced MG proliferation (Todd et al., 2015; Sardar Pasha et al., 2017). We hypothesized and confirmed that HBEGF, LPS, and the solvents DMSO and ethanol indeed show no, or only very low, changes, indicating that MROs at the age studied here are sufficiently stable to maintain homeostasis, are not excessively vulnerable, and are not already undergoing pathologic changes that can easily be augmented.

To conclude, we show that MROs provide a model system for experimentally inducible pathologies with different phenotypes useful for research into retinal degeneration and regeneration. Our data give an insight into the MRO system’s competence, providing starting points for further optimization. We show that combined HBEGF-TNF application is sufficient to induce a complex pathology in MROs, providing a model which is potentially relevant for advanced AMD and other pathologies. Our pharmacological data show that MROs provide mechanistic access, and a complete prevention of a complex pathology, highlighting the possibility of future broad-spectrum therapeutic treatments.

## Data availability statement

The original contributions presented in this study are included in the article/Supplementary material, further inquiries can be directed to the corresponding author. The in-house Fiji macro developed for cell displacement analysis of VSX2 cells was deposited at Zenodo repository with the identifiers, together with related resources and example files (https://doi.org/10.5281/zenodo.5188650).

## Author contributions

MV and MK contributed to the conceptualization, manuscript, and original draft. MK, MV, FW, PC, EK, and TK contributed to the manuscript and review and editing. MV, FW, and CK performed the MRO experiments and investigations. MV, FW, AS, CD, LE, CK, MK, and TK analyzed the data. AS and MV performed the western blot analysis. VIA prepared the microglia. TK performed the electron microscopy imaging. PC and MM built and established the blue-light setup, and methodology. MK, TK, and EK contributed to the funding acquisition. All authors contributed to the article and approved the submitted version.
