# Supplementary material for: Modeling inducible neuropathologies of the retina with differential phenotypes in organoids
Source: Front Cell Neurosci. 2023 May 5;17:1106287. doi: 10.3389/fncel.2023.1106287 (PMC10196395; doi:10.3389/fncel.2023.1106287)
Supplement: Supplementary file 1 [file Data_Sheet_1.DOCX]

Supplementary Material

# Supplementary Data

**Supplementary Experimental Procedures**

**Experimental design of MRO treatments: Inducible neuropathologies**

To establish a mouse retinal organoid (MRO) pathology model, we tested 16 challenges that might induce neuronal degeneration and/or glial pathology based on previous studies in animals or other models (Supplementary Table 1), including chemical, physical, and cell functional manipulations. We quantitatively assessed cell death (TUNEL assay), proliferation (KI67), and gliosis (GFAP) by histology 5 days after the challenges to identify potential hits. 16 pathology-inducing challenges were individually applied to 20-day-old MROs either once on day zero, or daily for 5 days (Fig.1A-C; Table 1; Supplementary Table 1). A minimum of n≥5 organoids in N≥1 experiments were analyzed for each challenge. Untreated or appropriate solvent control organoids were always derived from the same batch (N) of differentiation as experimental (challenged) organoids. For details on the challenges see below.

*Stab wound injury:* A stab wound was induced by stabbing the MRO retinal epithelia in four different positions located on different sides of the organoid on D20 using a 27G blunt-end needle (outer diameter 0.413mm; inner diameter 0.21mm; needle wall thickness 0.102mm). Regions within and adjacent to the stab area were analyzed, as well as unstabbed controls.

*Blue-light damage:* For blue-light treatment, organoids were irradiated with intense blue light using a custom-built setup consisting of two high-power LEDs (Huey Jann HPB8b-48K5BF/WPCB) with a center wavelength of 465nm. The LEDs were mounted directly in the cell culture incubator and positioned in a way that they each illuminated two 10cm dishes. For improved irradiance homogeneity, each LED was equipped with a custom light shade. The working distance between the LEDs and the cell-culture dishes was approximately 50mm. Power came from an associated LED driver unit (Lumotech L05012) providing an LED current of 350mA. The irradiance in the center of the illuminated areas was measured and set to 3mW/cm^2^, which decreased from the illumination center to the edge by a factor of 2. MROs were exposed to several different durations of constant blue-light irradiation (0.5, 1, 4, 6, 24, and 48h) starting on D20. After the treatment, samples were cultured using standard conditions until D25.

*Microglial challenge:* For microglial coculture experiments, single MROs were transferred to individual wells of a 96-well low-adhesion plate (U-bottom, Lipidure Coat, NOF) on D20 and cultured with or without (controls) microglia. Primary microglial cells were derived from whole brains of adult C57BL/6J wild-type mice and in-vitro expanded as previously published (Moussaud and Draheim, 2010). Primary mouse microglial cells were resuspended in organoid medium and added to each MRO (well) at a density of 5×10^4^ cells/organoid. Microglia and organoids were cocultured in 200µl medium per well. 50% of the medium was replaced daily.

*Chemical challenges:*

For chemical challenges, the tested factors were directly applied to the culture medium.

Recombinant proteins human HBEGF (heparin-binding EGF-like growth factor), human CNTF (ciliary neurotrophic factor), and mouse TNF (tumor necrosis factor) (all R&D), as well as SuperFASLigand (a biomimic of FasL) and SuperKillerTRAIL (a biomimic ligand for the TRAIL receptor of TNF-related apoptosis-inducing ligand (TRAIL)) (both Enzo Life Sciences, and both with enhanced stability) were added to the culture medium daily from D20 to D25 at a final concentration of 50ng/ml. HBEGF and TNF were also tested in combination (50ng/ml of each applied daily). Recombinant proteins were dissolved in sterile water (cell-culture grade), and an equal amount of water (0.5µl/ml) was added to solvent controls.

Smoothened agonist (SAG, Enzo Life Sciences) was added from D20 at each medium change (D20, D22, D24) at a final concentration of 0.25µM; MROs were analyzed on D25. SAG was dissolved in sterile DMSO. An equal amount of DMSO (0.25µl/ml) was applied to solvent controls. SAG was also tested together with HBEGF (SAG 0.25µM at medium change and HBEGF 50ng/ml daily).

L-glutamate and adenosine 5′-triphosphate (ATP) (both Sigma-Aldrich) were added to the culture medium from D20 to D22 at a final concentration of 1mM, then organoids were transferred back to normal culture medium until D25. Glutamate and ATP working stock solutions were directly prepared in culture medium, the pH was adjusted to 7.2.

The PDE6 inhibitor Zaprinast (Tocris) was added to the culture medium daily from D20 at a final concentration of 10µM, and MROs were analyzed on D25. Zaprinast was dissolved in sterile DMSO, and an equal amount of DMSO (1µl/ml) was applied to solvent controls.

The opsin agonist ß-ionone (Sigma-Aldrich) was added to the culture medium daily from D20 at a final concentration of 100µM, and MROs were analyzed on D25. ß-ionone was diluted in sterile ethanol, and an equal amount of ethanol (0.25µl/ml) was applied to solvent controls.

Lipopolysaccharides (LPS, Sigma-Aldrich) were added to the culture medium daily from D21 at a final concentration of 2µg/ml, and MROs were analyzed on D25. LPSs were dissolved in sterile water (cell-culture grade), and an equal amount of water (2µl/ml) was applied to solvent controls.

*Background information for neuronal damage and cell death-inducing challenges:*

Eight pathology-inducing challenges (Table 1; Supplementary Table 1) were explored. (1) Injuries penetrating and perforating the eye may result in severe vision loss or loss of the eye (Feldman et al.). Stab-wound injuries to the eye and retina are seen in emergency rooms due to knife assaults or household/work accidents, or may occur as iatrogenic penetration, e.g., from intravitreal injections (Senut et al., 2004; Sanchez-Lopez et al., 2005). (2) Excessive light exposure, specifically blue light, is known to cause severe retinal damage (Gottsch et al., 1993; Grimm et al., 2001; Laabich et al., 2006; Geiger et al., 2015; Calzia et al., 2016; Kim et al., 2016; Xia et al., 2019) and may involve degeneration of photoreceptor (PR) and inner retinal neurons, Müller glia (MG), and ganglion cells (Iandiev et al., 2008). (3) The opsin agonist ß-ionone is known to induce cell death in rod PRs in mature animal retinas (Alfinito and Townes-Anderson, 2002; Wang et al., 2012). (4) Glutamate excitotoxicity is the predominant cause of cerebral degeneration, and is known to induce ganglion and amacrine cell damage in the immature and mature retina (Olney, 1969; Xin et al., 2007; Mitori et al., 2016). (5) More recently, ATP has been shown to cause PR degeneration, either in combination with reactive gliosis or regeneration (Resta et al., 2007; Puthussery and Fletcher, 2009; Lu et al., 2015; Medrano et al., 2020; Brandli et al., 2021). ATP and glutamate may have a role in retinal degenerative diseases (Olney, 1982; Fletcher et al., 2019; Fletcher, 2020). (6) TRAIL is known to induce cell death, and has been associated with retinal pathology and reactive glia (Lee et al., 2002; Cantarella et al., 2007; Herrero-Martin et al., 2009; Chien and Dix, 2012). (7) Fas ligand (FASL) triggers apoptosis of Fas+ cells, including PRs in culture and, following retinal detachment, in vivo (Dunaief et al., 2002; Gregory et al., 2011; Matsumoto et al., 2015), indicating that some level of damage might be required for FASL-induced cell death. (8) Zaprinast, a pharmacological inhibitor of phosphodiesterase-6 (PDE6) has been shown to cause PR cell death via accumulation of cGMP-mimicking pathology upon PDE6 mutation in rods linked to congenital stationary night blindness (Vallazza-Deschamps et al., 2005; Martinez-Fernandez de la Camara et al., 2013; Sahaboglu et al., 2013; Wang et al., 2017).

*Background information for glial and complex retinal pathology-inducing challenges:*

The following eight challenges were studied (Fig.1A, C; Table 1; Supplementary Table 1). (1) CNTF is a well-known regulator of MG function which may induce reactive gliosis, and is expressed by MG upon reactive gliosis. MG-derived CNTF may provide neuroprotection for PRs, but also induce inflammation, as well as modulate MG proliferation and thus MG-derived neuronal regeneration, depending on the retinal state and animal model (Kassen et al., 2009; Xue et al., 2011; Wen et al., 2012; Todd et al., 2016). (2) Activation of SHH signaling in the postmitotic retina and in brain astrocytes has previously been associated with gliosis, and glial proliferation and regeneration (Wan et al., 2007; Todd and Fischer, 2015; Kaur et al., 2018; Thomas et al., 2018): Here we explored this in MROs by applying the SHH-signaling activator SAG (Chen et al., 2002). (3) HBEGF and (4) TNF, two factors known to contribute to retinal diseases (Nakazawa et al., 2011; Inoue et al., 2013; Sato et al., 2013; Cuenca et al., 2014; Sudharsan et al., 2017; Kim et al., 2018; Lyu et al., 2019; Saddala et al., 2019; Voigt et al., 2019), may regulate reactive gliosis and neuroprotection (Cuenca et al., 2014; Clarke et al., 2018; Dolz-Marco et al., 2018; Li et al., 2018; Kuchroo et al., 2021). Both factors are each also thought to be sufficient to induce MG proliferation and neuronal regeneration by activating MG reprogramming, at least in healthy zebrafish (Karl et al., 2008; Wan et al., 2012; Conner et al., 2014), whereas HBEGF-stimulated MG proliferation in rodents occurs only upon retinal damage (Karl et al., 2008; Sardar Pasha et al., 2017). To assess any synergistic function of (5) HBEGF and SHH-signaling, as well as (6) HBEGF and TNF (HT), we also applied these combinations. We recently showed that HT is sufficient to induce a complex pathology in human retinal organoids (Volkner et al., 2022). (7) Lipopolysaccharides (LPS) are known to induce pro-inflammatory genes in microglia, various changes in the retina, and retinal pigment epithelial cells opposed to the neural retina in vivo (Bian et al., 2016; Augustine et al., 2019). LPS stimulation causes closer interactions with MG when there is an inflammatory response, but no GFAP expression in MG in a healthy retina (Kassen et al., 2009; Dharmarajan et al., 2017; Augustine et al., 2019; Jia et al., 2019). However, systemic LPS stimulation accelerates PR degeneration and increased microglial numbers in a rat model of IRD (Noailles et al., 2018), and local LPS application increases neurodegeneration in adult retina in explant culture (Ghosh et al., 2018). Thus, we investigated whether LPSs has a pathologic effect in MROs with all major retinal cell types, but with no microglia. (8) Finally, we also applied primary mouse brain-derived microglia in coculture to MROs (Supplementary Fig. 1D-F). In the postmitotic retina in vivo, microglia are maintained and localized in the synaptic layers, and may contribute to pathologies in beneficial and detrimental ways: Microglia-derived factors may cause retinal neurodegeneration, activate specific reactive gliosis programs (Liddelow et al., 2017), migrate to pathologic sites, and phagocytize damaged cells to counteract degeneration (Akhtar-Schafer et al., 2018).

**Experimental design of MRO treatments:**

*HT application to MROs:*

In brief, organoids were differentiated until D20 using the standard culture conditions described in the Material and Methods section of the manuscript. From D20, recombinant human HBEGF and recombinant mouse TNF (HT) (both R&D) were applied to the culture medium daily, each at a final concentration of 50ng/ml. Controls received only solvent (cell-culture-grade water, 1µl/ml daily). Samples were taken at indicated days after the start of HT treatment, and processed for histological analysis. N≥3 independent experiments with n≥5 organoids per experiment were analyzed per timepoint, and untreated solvent controls were run in each experiment.

To determine if HT maintains or changes in phenotype beyond the 5 days of HT treatment (Supplementary Fig.3), MRO were also analyzed at day 27, 29, and 31 upon its removal (HT treated daily from day 20 to 25). To determine if HT treatment induces a pathology not only in 20-day-old MROs upon reaching postmitosis (Völkner et al. 2021), but also during development (Supplementary Fig.5), we tested it on MROs differentiated until mid retinogenesis (D15) as described in the Material and Methods section of the manuscript and reported previously (Völkner et al. 2016). From D15 to D18, HBEGF and TNF were applied daily either separately or in combination, both at a final concentration of 50ng/ml. In comparison to HT, we applied DAPT (Sigma-Aldrich) daily from D16 to D18 at a concentration of 10µM to inhibit Notch signaling, which is known to interrupt retinogenesis by inducing neuronal differentiation and depletion of retinal progenitors (Völkner et al. 2016). DAPT thus served here as a positive control. To monitor cell proliferation, MROs received an EdU pulse (1µM, Invitrogen) 2 hours before fixation at D18. To monitor retinogenesis, we used immunostaining to analyze ASCL1, which is expressed in a subpopulation of neurogenic retinal progenitors.

For microglial coculture experiments in HT-treated and control MRO, single MROs were transferred to individual wells of a 96-well low-adhesion plate (U-bottom, Lipidure Coat, NOF) in 200µl medium on D20. MRO were treated daily with HT from D20, both at a final concentration of 50ng/ml. Controls received only solvent (water). On D21, primary mouse microglial cells were added half of the HT-treated and control MROs at a density of 5×10^4^ cells/organoid (well). Primary microglial cells were derived from whole brains of adult C57BL/6J wild-type mice and in-vitro expanded as previously published (Moussaud and Draheim, 2010). 50% of the medium was replaced daily.MROs were processed for immunohistochemistry on D25 (N=1 independent experiments with n=4-5 organoids per condition).

*Application schemes of pharmacological inhibitors:*

To analyze the underlying signaling pathways of the HT phenotype, pharmacological inhibitor experiments were performed. MROs were differentiated until D20 using the standard protocol and HT was applied daily at a final concentration of 50ng/ml each from D20 to D25. The pharmacological signaling inhibitors were applied starting 7h before HT treatment, and then added daily until D25 at the same time as HT. The following inhibitors were used: U0126 (MEK inhibitor, Calbiochem, Merck) at a final concentration of 10µM (stock solution 20mM in DMSO); NSC625987 (CDK4 inhibitor, Tocris) at a final concentration of 10µM (stock solution 20mM in DMSO); Y‑27632 (ROCK inhibitor, Tocris) at a final concentration of 10µM (stock solution 20mM in cell culture-grade water); BAY-11-7082 (NFkB inhibitor, Enzo Life Sciences) at a final concentration of 0.5µM (stock solution 1mM in DMSO). Controls received equal amounts of solvents (DMSO or water). Samples were taken at D25 and processed for immunohistochemistry. N=3 independent experiments with n≥10/N MROs were analyzed per treatment condition.

*Background information on the selected signaling pathways of interest:*

ERK/MAPK: The function of the mitogen-activated protein kinase (MAPK) extracellular signal-regulated kinase (ERK), has been linked to PR cell survival in experimental, IRD, and AMD models (Kinkl et al., 2001; German et al., 2006; Swain et al., 2007; Kyosseva, 2016; Samardzija et al., 2021). Further, ERK/MAPK and EGF receptor expression are upregulated in MG undergoing reactive gliosis after retinal damage and disease (Geller et al., 2001; Kinkl et al., 2001; Kase et al., 2006; Kassen et al., 2009; Groeger et al., 2012; Sardar Pasha et al., 2017). MAPK inhibition may reduce MG functions, e.g., reactive gliosis and neuroprotection (Hauck et al., 2006; Inoue et al., 2013; Gao et al., 2017). Experimental application of HBEGF or EGF stimulates MG proliferation in damaged juvenile and adult mouse retinas (Karl et al., 2008; Loffler et al., 2015). HBEGF is even sufficient to induce MG proliferation, and thus retinal regeneration, in zebrafish; MG proliferation requires MAPK activation (Kase et al., 2006; Fischer et al., 2009; Kassen et al., 2009; Wan et al., 2012; Ueki and Reh, 2013; Wan et al., 2014; Todd et al., 2015). Of note, longer-term MAPK inhibitor treatment of patients with non-ocular cancer may cause retinal pathology (Tyagi and Santiago, 2018), and this might be due to its inhibitory function on MG. Application of MEKi (UO126) to HT-induced human retinal organoids prevent the complex pathology (Volkner et al., 2022).

NFkB: The nuclear factor-kappa B (NFkB) signaling pathway regulates cell survival, cell proliferation, and inflammation, and is activated by oxidative stress and proinflammatory cytokines. NFkB signaling may be activated upon retinal damage in MG and PRs: Loss of NFkB may increase PR degeneration (Krishnamoorthy et al., 1999; Wu et al., 2002; Yang et al., 2007). Further, NFkB inhibition enhances the induction of MG proliferation and thus regeneration or reactive gliosis, and activation suppresses it, possibly depending on the animal model and disease conditions (Lebrun-Julien et al., 2009; Sifuentes et al., 2016; Jia et al., 2019; Li et al., 2020; Palazzo et al., 2020).

ROCK: Inhibition of rho-associated kinase (ROCK) signaling may reduce reactive gliosis of MG in mammalian disease models, including a decrease in GFAP (Tura et al., 2009; Alt et al., 2013; Mohammad et al., 2018), while the absence of ROCK causes a reduction in MG cell-cycle re-entry in the regenerating zebrafish retina. In addition, at least in regenerating and developing zebrafish, ROCK regulates MG proliferation via an actin-myosin-mediated process called interkinetic nuclear migration (Nagashima et al., 2013; Lahne et al., 2015). However, it is not known if this process might also regulate cell displacement in degenerating retina, as described as part of retinal remodeling (Jones et al., 1995; Jones et al., 2016). Further, applying selective inhibitors for ROCK1 and ROCK2, like the clinically-used Fasudil, as well as ROCKi (Y-27632), stabilize PR synapses in pathologic models, and thereby support PR survival (Zhang et al., 2015; Townes-Anderson et al., 2017; Mohammad et al., 2018; Wang et al., 2019; Halasz et al., 2021; Townes-Anderson et al., 2021).

CDK4: Cyclins and cyclin-dependent kinases (CDK) act together as a mitogenic sensor, providing a link between the environment and the cell-cycle machinery. Synthesis of Ccnd1 drives cell-cycle progression by interacting with positive cell-cycle regulators like cyclin-dependent kinase 4 (CDK4) to form an active complex. This complex phosphorylates regulatory substrates, which are rate-limiting steps in cell-cycle progression. Ccnd1 becomes downregulated at the end of retinogenesis, and cell-cycle inhibitors, like the cyclin-dependent kinase inhibitors Cdkn1a and Cdkn1b, are upregulated in postmitotic retinal neurons and glia, which inhibit cell-cycle progression (Dyer and Cepko, 2001). Conversely, inactivation of the cyclin-dependent kinase inhibitor 1B (Cdkn1b, p27) results in pathologic MG cell-cycle re-entry and proliferative gliosis (Dyer and Cepko, 2000; Levine et al., 2000; Vazquez-Chona et al., 2011), and Cdkn1a (p21) might also be involved (Ueki et al., 2012). Activation of cell-cycle machinery has also been observed in some models of PR degeneration (Zencak et al., 2013; Arsenijevic, 2016). Here, we inhibited CDK4 (CDK4i; NSC625987), which has previously been thought to be involved in MG proliferation (Yoshida et al., 2004; Kase et al., 2006; Karl et al., 2008; Loffler et al., 2015; Sang et al., 2015; Schafer and Karl, 2017).

**Histological analysis, imaging, and quantification methods:**

*Immunohistochemistry:*

For immunohistochemistry, MROs were fixed in 4% PFA in PBS, cryoprotected in a graded series of sucrose solutions, and embedded in tissue-freezing medium (Jung). Organoids were sectioned at 12μm thickness using a Leica CM3050 S cryostat, mounted on Superfrost slides (Thermo Scientific) and stored at −80°C. Sections were washed in PBS for 15min; if necessary, antigen retrieval was performed via citrate (10mM sodium citrate, pH 6.0, 30min at 70°C). The tissue was treated for 30min at RT in blocking solution (0.5% BSA and 0.3% Triton-X-100 in PBS), then incubated in the primary antibodies (48h, 4°C). The tissue was washed in PBS (3×10min) and species-specific secondary antibodies conjugated to fluorophores (488, Cy3, 649; Dianova, 1:1000) were applied for 1h at RT. Nuclei were stained using DAPI (AppliChem). The tissue was washed again in PBS and coverslipped using Fluoromount-G (Southern Biotechnology). TUNEL assay for cell death analysis was performed before primary antibody incubation using In Situ Cell Death Detection Kit TMR red (Sigma-Aldrich, Roche products) according to the manufacturer’s instructions. Filamentous actin was visualized using Phalloidin488 (PHAL, Invitrogen) staining (1:500, 15 min at RT) after secondary-antibody incubation. EdU detection was performed using Click-iT® EdU Alexa Fluor Imaging Kits (Invitrogen) according to the manufacturer’s instructions. The primary antibodies used in this study are listed in Supplementary Table 5.

*Imaging and quantitative analysis of MRO cellular changes:*

Samples were imaged on a Zeiss ApoTome2 microscope. Random regions of interest (ROIs) of 100μm width along the outer (apical) surface were used for cell counts and co-localization analysis. The x-axis of each ROI was positioned radially to the organoid center, with the y-axis aligned perpendicular to the organoid surface. The dimensions of the ROI was set to include the entire epithelial width. ROI images are z-axis projections of 5×1μm, i.e., 5 planes, 1μm apart acquired in Apotome mode using a 20× Plan-Apochromat objective. For cell counts, images were 3D reconstructed (maximum intensity projection) and counted using the cell counter tool in the Fiji software (http://fiji.sc) (Schindelin et al., 2012). For quantitative analysis of GFAP, RCVRN, and DAPI staining, images were automatically thresholded in Fiji (using mean threshold mode) and the pixel area above the threshold was measured. RCVRN pixel area was normalized to DAPI pixel area in each ROI. OPN1SW+ cells were counted from entire central organoid sections and normalized to organoid circumference. Organoid circumference and epithelial thickness were measured on microscopic images of entire central organoid sections or ROIs, respectively, using Fiji; for all parameters, the apical organoid boundary was defined using DAPI staining, and the basal boundary by VSX2 staining. To assess the loss of layering, each organoid was scored on multiple (6-8) serial sections (each 120μm apart) for the presence of a retinal-layer-like structure, characterized by an epithelial tissue organization with distinct nuclear layers (DAPI): An apical layer with multiple rows of PR cells (RCVRN+) followed by a layer with very few RCVRN+ but many VSX2+ cells. For microglia co-culture experiments, the localization of microglia cells (IB4+) within the MROs was quantified on entire MRO central sections and normalized to MRO circumference. The localization of microglia within the retinal epithelium, apical boundary defined by DAPI and basal boundary by VSX2, and the inner MRO, defined as the region basal of the VSX2+ cells, was counted separately. In addition, some microglia cells were also found on the outer surface of the MRO, i.e. apically outside if the retinal epithelium, which was counted separately too.

*Quantitative analysis of cell displacement:*

Cell displacement analyses of VSX2+ cell nuclei were performed on microscopic ROI images (see above). A Fiji (http://fiji.sc) software macro (cell displacement analysis (0.1.0), Zenodo, https://doi.org/10.5281/zenodo.5188650) was used to automatically threshold images and detect the precise position (x/y coordinates) of positive signals. Local maxima detection (3D maxima finder, 3D ImageJ Suite) was used to determine the position of individual VSX2+ nuclei. For visualization purposes, each ROI was divided into 10 equally sized segments from the apical to the basal organoid surface (along the y-axis), and the relative number of local maxima (VSX2+ cell nuclei) detected was calculated for each segment.

*Histology of methacrylate resin sections:*

The histology of MROs was studied uning a previously described methodology (Volkner et al., 2019). Briefly, MROs were fixed in modified Karnovsky’s fixative (2% glutaraldehyde, 2% paraformaldehyde in 50mM HEPES) overnight at 4°C (Kurth et al., 2010). Samples were washed, postfixed in 1% OsO_4_/PBS, washed again, and dehydrated in a graded series of ethanol. Samples were infiltrated in Technovit 7100 and embedded. 2μm sections were cut using a rotary microtome. Sections were stained with 1% toluidine blue/0.5% borax and imaged using Keyence Biozero 8000 fluorescence microscope.

*Scanning electron microscopy (SEM):*

SEM of MROs was performed as previously described (Volkner et al., 2021). Briefly, MROs were fixed in modified Karnovsky’s fixative (2% glutaraldehyde, 2% paraformaldehyde in 50mM HEPES). After washing in HEPES and PBS, samples were postfixed in 1% OsO_4_/PBS, washed in PBS and water, and dehydrated in a graded ethanol series. Samples were critical-point dried using the Leica CPD 300 (Leica Microsystems, Vienna, Austria). Dried whole MROs were mounted on 12mm aluminum stubs; some MROs were manually dissected using a scalpel. Thereby samples preferentially break apart between cell borders. Finally, samples were sputter-coated with gold using the Baltec SCD 050 (Leica) and analyzed with a JSM 7500F cold field emission SEM (JEOL) at 8mm working distance and 5–10kV acceleration voltage using the lower secondary electron detector.

*Transmission electron microscopy (TEM):*

TEM of MROs was performed as previously described (Volkner et al., 2019; Volkner et al., 2021). Briefly, MROs were fixed in 4% formaldehyde (prepared from paraformaldehyde prills) in 100mM phosphate buffer and dissected for different applications. Samples selected for resin embedding and TEM were postfixed in modified Karnovsky’s fixative (2% glutaraldehyde, 2% paraformaldehyde in 50mM HEPES) overnight at 4°C (Kurth et al., 2010). Samples were washed and further postfixed in 2% aqueous OsO_4_ solution containing 1.5% potassium ferrocyanide and 2mM CaCl_2_. After washing, samples were incubated in 1% thiocarbohydrazide, washed again, and contrasted in osmium for a second time. After washing, samples were en-bloc contrasted with 1% uranyl acetate/water, washed again in water, dehydrated in a graded ethanol series, and infiltrated in the epon substitute EMBed 812. After embedding, samples were cured at 65°C overnight. Ultrathin sections were cut with a Leica UC6 ultramicrotome and collected on formvar-coated slot grids. Sections were stained with lead citrate (Venable and Coggeshall, 1965) and uranyl acetate, and imaged on a FEI Morgagni D268 (camera: MegaView III, Olympus) or a Jeol JEM1400 Plus (camera: Ruby, JEOL) both running at 80kV acceleration voltage.

**Protein preparation and western blot analysis:**

MROs were treated with and without HT as described above and individual MROs (HT-treated and controls) were collected at 10min and 8h after the start of HT treatment. MROs were snap frozen in liquid nitrogen and immediately stored at −80°C. Samples were lysed on ice for 20min in 1×LDS sample buffer (Thermo Scientific) supplemented with 3% b-mercaptoethanol (Sigma), protease inhibitors (cOmplete mini, Roche), and phosphatase inhibitors (PhosSTOP, Roche). Subsequently, samples were sonicated and clarified by centrifugation at 16,000×*g* for 10min at 4°C. Lysates were boiled for 5min and separated on 4-12% NuPage gels, transferred to polyvinylidene fluoride membranes (Millipore), and probed with primary antibody (see Supplementary Table 5). Immunoreactive bands were detected using donkey-anti-mouse or donkey-anti-rabbit secondary antibodies (Dianova) and SuperSignal West Dura Chemiluminescent Substrate (Thermo Scientific), then captured on Amersham Hyperfilm ECL (GE Healthcare). Immunoreactive bands were quantified using Fiji software (http://fiji.sc) . Signals of pERK, pSTAT3, pNFkB, and pAKT were normalized to tERK, and ratios were determined for individual MROs.

# Supplementary Figures and Tables

## Supplementary Figures

**Supplementary Fig1.** **Representative images of experimental challenges of MROs.**

Representative images of the analysis of the experimental challenges of MRO. MRO were challenged with treatments hypothesized to induce either neuronal damge (A-E) or gliosis (F-J) (see supplementary table 1, Supplementary Experimental Procedures for details). MRO tissue sections were labeled with GFAP, TUNEL, Ki67 to assess reactive gliosis, cell death, and proliferation respectively. Represenative images for the challenges not shown here are shown in Fig.2. See Fig.2A, E and Supplementary table 2 for quantitative data. Scale bar: 25µm.

**Supplementary Fig2.** **Additional data on experimental challenges of MROs.**

(A) Representative images and quantification of GFAP in MROs treated with blue light for different time intervals starting from day (D) 20 and analyzed on D25. Each circle represents one individual organoid (n) dereived from 1 independent experiment (N). Error bars: (SD). * p<0.05 (one-way ANOVA with Tukey’s posthoc test). (B) Immunostaining of aCASP3 (apoptosis marker) in MROs treated with blue light for different time intervals starting from day (D) 20 and analyzed on D25. (C) Representative image and quantitive analysis of cGMP in control (DMSO) and Zaprinast treated MRO. cGMP colocalizes with photoreceptor marker RCVRN. The dashed square indicates the region shown at higher magnification. Each circle represents one individual organoid (n) dereived from 1 independent experiment (N). Error bars: (SD). * p<0.05 (Students t-test). (D) Microglia coculture: The majority of IB4+ cells (microglia) in the MRO colabel for IBA1 in immunohistochemistry of MROs coculture with primary microglia from D20 to D23. (E) Quantification of the localization of the IB4+ cells within the MROs after coculture with primary microglia from D20 to D23. The majority are localized in the inner region of the organoid (inner RO), and some IB4+ cells are found in the outer nuclear-like layer (ONL) or on the outer surface of the MRO (surface). Data show mean of n=31 MRO derived from N=4 independent experiments. Scale bar: 25µm.

**Supplementary Fig.3. Supplementary data on photoreceptor degeneration and cell death in the HBEGF-TNF-induced pathologic model.**

(A) Representative images of RCVRN (photoreceptors), VSX2 (Müller glia), and DAPI staining in MROs treated with TNF only and HBEGF only from D20 to D25. (B) Histologic analysis of cone photoreceptor pathology in HBEGF-TNF (HT)-challenged MROs: Cone photoreceptor cells expressing S-opsin (OPN1SW) are present at low numbers in 25-day-old control (CTRL) MROs. A significant reduction was seen after 5 days of daily HT challenge (D20-D25); n=6/N with N=1. We previously reported that cones expressing M/L-opsin could not be detected in this MRO system (Völkner et al. 2021). (C-F) Representative images and quantitative analysis of immunostaining study on cell death-related markers: (C) TUNEL assay and activated caspase 3 (aCASP3) at D25; (D) cGMP at D25; and (F) RIPK3 and PAR at D22 and D25 on histological sections of MROs treated with and without HT. n=5/N, N=3 per variable. (E) cGMP+ cells colocalize with RCVRN (photoreceptors). The dashed square indicates the region shown at higher magnification. * Statistically significant (Supplementary Table 4: Detailed data and p-values). Scale bar: 25µm.

## Supplementary Fig.4. Dynamics of the HBEGF-TNF-induced pathologic MRO model.

HT-challenged MROs rapidly develop a pathologic phenotype within 5 days, and do not show epithelial thinning of the retina although there is photoreceptor loss. (A) Experimental paradigm. MROs were HT challenged on D20-D25 and analyzed at several later timepoints to evaluate longer-term effects. (B) Quantitative analysis of retinal thickness in MROs in controls (CTRL) and after HT challenge. The retinal thickness of CTRL or HT-challenged MROs did not change between D25 and D31. Retinal thickness of HT-challenged MROs was at both timepoints higher than in CTRL. (C) Representative microscopic images after immunostaining for photoreceptors (RCVRN), Müller glia (VSX2, SOX8, RLBP1), gliosis (GFAP), and proliferation (KI67). Gliosis levels slightly increased in CTRL over time, while gliosis levels and Müller glia displacement were similar in HT-challenged MROs across all timepoints. The number of proliferating cells in HT-challenged MROs declined from D25 to D31. (D) Representative microscopic images of MROs treated with or without HBEGF-TNF (HT) for 5 days (day 20 to 25): MRO crossections were immunostained for markers labeling photoreceptors (RCVRN), Müller glia and some bipolars (VSX2), and cell nuclei (DAPI). Graphs: Each circle represents one individual MRO (n) based on N=1 independent experiment (n=4-5/N). Error bars represent standard deviations (SD). * Statistically significant. Scale bars: (C) 25µm; (D) 100µm.

**Supplementary Fig.5.** **Microglia coculture with HBEGF-TNF-challenged MROs.**

Data related to Fig.4F. Representative images of cell proliferation in the retina in MROs challenged with and without HT and with and without microglial coculture. Cell proliferation was monitored by immunostaining for KI67 and microglia by IB4 staining in MRO sections. Scale bar: 25µm.

**Supplementary Fig.6.** **HBEGF-TNF challenge of developing MROs.**

(A) Experimental paradigm. MROs were challenged during retinogenesis by treatment with HBEGF only, TNF only, or a combination (HT) from D15 to D18. Application of the Notch inhibitor DAPT served as a positive control for any effect on cell proliferation. Proliferating cells were labeled by an EdU pulse (2h) prior to fixation. (B) Representative brightfield images at D18. (C) Representative microscopic images after immunostaining and (D) quantitative analyses: Unlike in controls (CTRL), DAPT-treated MROs showed no cell proliferation (EdU) or the neurogenic marker ASCL1, while the number of photoreceptor cells (CRX), ectopic photoreceptors, and Müller glia (SOX2) was increased. Separate HBEGF or TNF, or combined HT challenge had no effects. Gliosis (GFAP) was absent, and cell death (TUNEL) was comparably low in all groups. Graphs: Each circle represents one individual MRO (n) based on N=1 independent experiment (n=5/N). Error bars represent standard deviations (SD). * Statistically significant. Scale bar: 500µm (B), 25µm (C, applies to all panels).

**Supplementary Fig.7.** **Western blot analysis of selected signaling pathways in the HBEGF-TNF-induced pathology model**.

Complete western blot data related to Fig.7. Western blot analysis of whole MRO lysates collected after 10min and 8h of HT treatment compared to solvent controls (n=4 MROs per variable) to determine phosphorylation of ERK1/2 (pERK), AKT (pAKT), STAT3 (pSTAT3), and NFkB (pNFkB). Immunoreactive bands were quantified using Fiji software (http://fiji.sc) , and pERK, pSTAT3, pNFkB, and pAKT signals were normalized to total ERK (tERK); ratios were determined for individual MROs.

## Supplementary Tables

**Supplementary Table 1: Overview of the experimental challenges used to induce neuropathologies.**

Data related to Fig.1. Description, objectives, experimental design, and references for the experimental challenges applied, as well as phenotypes observed in the mouse retinal organoids. Most of the experimental challenges presented have been used widely before, but some are less well established. We apologize to those colleagues whose work we did not cite due to space limitations. For technical details see Supplementary Experimental Procedures. Abbreviations: n.d.: not determined; MRO: mouse retinal organoid; D: day of culture;AMD: age-related macular degeneration; IRD: inherited retinal dystrophy; DAMP: damage-associated molecular patterns (e.g., released from dying cells); CNTF: ciliary neurotrophic factor; SAG: smoothened agonist; SHH: sonic hedgehog; DMSO: dimethyl sulfoxide; FasL: Fas ligand;HBEGF: heparin-binding EGF-like growth factor;LPS: lipopolysaccharides; TNF: tumor necrosis factor alpha; TRAIL: TNF-related apoptosis-inducing ligand; MG: Müller glia; PR: photoreceptor; RGC: retinal ganglion cell; RPE: retinal pigment epithelium; dro: drosophila; zf: zebrafish; xp: xenopus; s: salamander; ch: chicken; qu: quail; m: mouse; r: rat; rb: rabbit; d: dog; p: pig; pr: primate; h: human.

References for the challenges given in the table:

**HBEGF+TNF** (Janes et al., 2006; Kovacs et al., 2015; Volkner et al., 2022)

**TNF** (Cotinet et al., 1997; Yuan and Neufeld, 2000; Nakazawa et al., 2006; Giaume et al., 2007; Lebrun-Julien et al., 2009; Fernandez-Bueno et al., 2013; Nelson et al., 2013; Cuenca et al., 2014; Faber et al., 2015; Appelbaum et al., 2017; Galan et al., 2017; Huang et al., 2017; Tonade et al., 2017; Xie et al., 2017; Volkner et al., 2022)

**HBEGF** (Hollborn et al., 2005; Karl et al., 2008; Wan et al., 2012; Todd et al., 2015; Volkner et al., 2022)

**CNTF** (Wen et al., 1995; Fischer et al., 2004a; Fischer et al., 2004b; Kassen et al., 2009; Xue et al., 2011)

**SAG** (Spence et al., 2004; Wan et al., 2007; Todd and Fischer, 2015; Thomas et al., 2018)

**SAG+HBEGF** (Bhatia et al., 2009; Gotschel et al., 2013)

**Microglia** (Roque et al., 1999; Fischer et al., 2014; Zhao et al., 2015; White et al., 2017)

**LPS (endotoxin)** (Jacquemin et al., 1996; Takeda et al., 2002; Arai et al., 2003)

**Stab wound** (Senut et al., 2004; Sanchez-Lopez et al., 2005; Kanamori et al., 2012)

**Blue light** (Gottsch et al., 1993; Grimm et al., 2001; Calzia et al., 2016; Kim et al., 2016)

**β-ionone** (Alfinito and Townes-Anderson, 2002; Wang et al., 2012)

**Glutamate** (Olney, 1969; Xin et al., 2007; Mitori et al., 2016)

**ATP** (Resta et al., 2007; Puthussery and Fletcher, 2009; Lu et al., 2015)

**FasL** (Dunaief et al., 2002; Gregory et al., 2011; Matsumoto et al., 2015)

**TRAIL** (Cantarella et al., 2007; Chien and Dix, 2012)

**Zaprinast** (Vallazza-Deschamps et al., 2005; Martinez-Fernandez de la Camara et al., 2013; Sahaboglu et al., 2013; Wang et al., 2017)

**DMSO** (Galvao et al., 2014)

**Ethanol** (Stromland and Pinazo-Duran, 2002; Sancho-Tello et al., 2008; Miao et al., 2013)

**Supplementary Table 2: Summary of quantitative data acquired for the experimental challenges.**

Summary of quantitative data shown in Fig.2. For technical details on the experimental challenges see Supplementary Table 1 and Supplementary Experimental Procedures. N≥1 experiments; n≥5/N organoids) were quantified for each challenge. Controls were run independently in each experiment. p-values are shown. Abbreviations: n.d.: not determined; ROI: region of interest.

**Supplementary Table 3: Summary of quantitative data for the HT-MRO model and comparison to the HT-HRO model.**

Table sheet A: Summary of quantitative data shown in Fig.3 & 4 and Supplementary Fig.3. Mouse retinal organoids (MROs) have been treated with HBEGF-TNF (HT) and without (control, CTRL) from D20 to D25. Details on numbers of individual organoids (n) and independent experiments (N) analyzed, as well as p-values (Student’s t-test) are shown. Abbreviations: MRO: mouse retinal organoid; D: day; CTRL: control; HT: HBEGF-TNF; ROI: region of interest (see Supplementary Experimental Procedures); MG: Müller glia; PR: photoreceptor; n: number of individual organoids analyzed; N: number of independent experiments. Table sheet B: Comparison of the HT-induced pathology in MROs to the data published for HROs (Völkner et al. 2022).

**Supplementary Table 4: Summary of quantitative data for pharmacological study of the HT-MRO model.**

Overview of quantitative data shown in Fig.5. MROs were differentiated until D20 and then HT treatment was applied. Specific inhibitors of NFkB, ROCK, MEK, and CDK4 were applied starting 7h before HT addition and then throughout the HT treatment period at the same time as HT. MROs were analyzed at D25. N=3 independent experiments; n≥10/N MROs were analyzed and p-values (one-way ANOVA with Tukey’s posthoc test) are shown. Abbreviations: MRO: Mouse retinal organoid; CTRL: control; HT: HBEGF-TNF; ROI: region of interest (see methods); MG: Müller glia; PR: photoreceptor; n: number of individual organoids analyzed; N: number of independent experiments; n.s.: not statistically significant; vs: compared to; i: inhibitor.

**Supplementary Table 5: List of antibodies and in-situ assays used.**

Abbreviations: Catalogue number (Cat.no).

**Supplementary References:**

Akhtar-Schafer, I., Wang, L., Krohne, T.U., Xu, H., and Langmann, T. (2018). Modulation of three key innate immune pathways for the most common retinal degenerative diseases. *EMBO Mol Med* 10(10). doi: 10.15252/emmm.201708259.

Alfinito, P.D., and Townes-Anderson, E. (2002). Activation of mislocalized opsin kills rod cells: a novel mechanism for rod cell death in retinal disease. *Proc Natl Acad Sci U S A* 99(8)**,** 5655-5660. doi: 10.1073/pnas.072557799.

Alt, A., Hilgers, R.D., Tura, A., Nassar, K., Schneider, T., Hueber, A., et al. (2013). The neuroprotective potential of Rho-kinase inhibition in promoting cell survival and reducing reactive gliosis in response to hypoxia in isolated bovine retina. *Cell Physiol Biochem* 32(1)**,** 218-234. doi: 10.1159/000350138.

Appelbaum, T., Santana, E., and Aguirre, G.D. (2017). Strong upregulation of inflammatory genes accompanies photoreceptor demise in canine models of retinal degeneration. *PLoS One* 12(5)**,** e0177224. doi: 10.1371/journal.pone.0177224.

Arai, K., Wood, J.P., and Osborne, N.N. (2003). Beta-adrenergic receptor agonists and antagonists counteract LPS-induced neuronal death in retinal cultures by different mechanisms. *Brain Res* 985(2)**,** 176-186.

Arsenijevic, Y. (2016). Cell Cycle Proteins and Retinal Degeneration: Evidences of New Potential Therapeutic Targets. *Adv Exp Med Biol* 854**,** 371-377. doi: 10.1007/978-3-319-17121-0_49.

Augustine, J., Pavlou, S., Ali, I., Harkin, K., Ozaki, E., Campbell, M., et al. (2019). IL-33 deficiency causes persistent inflammation and severe neurodegeneration in retinal detachment. *J Neuroinflammation* 16(1)**,** 251. doi: 10.1186/s12974-019-1625-y.

Bhatia, B., Singhal, S., Lawrence, J.M., Khaw, P.T., and Limb, G.A. (2009). Distribution of Muller stem cells within the neural retina: evidence for the existence of a ciliary margin-like zone in the adult human eye. *Exp Eye Res* 89(3)**,** 373-382. doi: 10.1016/j.exer.2009.04.005.

Bian, M., Du, X., Cui, J., Wang, P., Wang, W., Zhu, W., et al. (2016). Celastrol protects mouse retinas from bright light-induced degeneration through inhibition of oxidative stress and inflammation. *J Neuroinflammation* 13**,** 50. doi: 10.1186/s12974-016-0516-8.

Brandli, A., Dudczig, S., Currie, P.D., and Jusuf, P.R. (2021). Photoreceptor ablation following ATP induced injury triggers Muller glia driven regeneration in zebrafish. *Exp Eye Res* 207**,** 108569. doi: 10.1016/j.exer.2021.108569.

Calzia, D., Panfoli, I., Heinig, N., Schumann, U., Ader, M., Traverso, C.E., et al. (2016). Impairment of extramitochondrial oxidative phosphorylation in mouse rod outer segments by blue light irradiation. *Biochimie* 125**,** 171-178. doi: 10.1016/j.biochi.2016.03.016.

Cantarella, G., Bucolo, C., Di Benedetto, G., Pezzino, S., Lempereur, L., Calvagna, R., et al. (2007). Protective effects of the sigma agonist Pre-084 in the rat retina. *Br J Ophthalmol* 91(10)**,** 1382-1384. doi: 10.1136/bjo.2007.118570.

Chen, J.K., Taipale, J., Young, K.E., Maiti, T., and Beachy, P.A. (2002). Small molecule modulation of Smoothened activity. *Proc Natl Acad Sci U S A* 99(22)**,** 14071-14076. doi: 10.1073/pnas.182542899.

Chien, H., and Dix, R.D. (2012). Evidence for multiple cell death pathways during development of experimental cytomegalovirus retinitis in mice with retrovirus-induced immunosuppression: apoptosis, necroptosis, and pyroptosis. *J Virol* 86(20)**,** 10961-10978. doi: 10.1128/JVI.01275-12.

Clarke, L.E., Liddelow, S.A., Chakraborty, C., Munch, A.E., Heiman, M., and Barres, B.A. (2018). Normal aging induces A1-like astrocyte reactivity. *Proc Natl Acad Sci U S A* 115(8)**,** E1896-E1905. doi: 10.1073/pnas.1800165115.

Conner, C., Ackerman, K.M., Lahne, M., Hobgood, J.S., and Hyde, D.R. (2014). Repressing notch signaling and expressing TNFalpha are sufficient to mimic retinal regeneration by inducing Muller glial proliferation to generate committed progenitor cells. *J Neurosci* 34(43)**,** 14403-14419. doi: 10.1523/JNEUROSCI.0498-14.2014.

Cotinet, A., Goureau, O., Hicks, D., Thillaye-Goldenberg, B., and de Kozak, Y. (1997). Tumor necrosis factor and nitric oxide production by retinal Muller glial cells from rats exhibiting inherited retinal dystrophy. *Glia* 20(1)**,** 59-69.

Cuenca, N., Fernandez-Sanchez, L., Campello, L., Maneu, V., De la Villa, P., Lax, P., et al. (2014). Cellular responses following retinal injuries and therapeutic approaches for neurodegenerative diseases. *Prog Retin Eye Res* 43**,** 17-75. doi: 10.1016/j.preteyeres.2014.07.001.

Dharmarajan, S., Fisk, D.L., Sorenson, C.M., Sheibani, N., and Belecky-Adams, T.L. (2017). Microglia activation is essential for BMP7-mediated retinal reactive gliosis. *J Neuroinflammation* 14(1)**,** 76. doi: 10.1186/s12974-017-0855-0.

Dolz-Marco, R., Balaratnasingam, C., Messinger, J.D., Li, M., Ferrara, D., Freund, K.B., et al. (2018). The Border of Macular Atrophy in Age-Related Macular Degeneration: A Clinicopathologic Correlation. *Am J Ophthalmol* 193**,** 166-177. doi: 10.1016/j.ajo.2018.06.020.

Dunaief, J.L., Dentchev, T., Ying, G.S., and Milam, A.H. (2002). The role of apoptosis in age-related macular degeneration. *Arch Ophthalmol* 120(11)**,** 1435-1442.

Dyer, M.A., and Cepko, C.L. (2000). Control of Muller glial cell proliferation and activation following retinal injury. *Nat Neurosci* 3(9)**,** 873-880. doi: 10.1038/78774.

Dyer, M.A., and Cepko, C.L. (2001). Regulating proliferation during retinal development. *Nat Rev Neurosci* 2(5)**,** 333-342. doi: 10.1038/35072555.

Faber, C., Jehs, T., Juel, H.B., Singh, A., Falk, M.K., Sorensen, T.L., et al. (2015). Early and exudative age-related macular degeneration is associated with increased plasma levels of soluble TNF receptor II. *Acta Ophthalmol* 93(3)**,** 242-247. doi: 10.1111/aos.12581.

Feldman, B.H., Shah, V.A., Murchison, A., Parker, P.R., Hsu, J., J.I., L., et al. (2021). American Academy of Ophthalmology, EyeWiki. Available: https://eyewiki.aao.org/Ocular_Penetrating_and_Perforating_Injuries [Accessed November 2021.].

Fernandez-Bueno, I., Garcia-Gutierrez, M.T., Srivastava, G.K., Gayoso, M.J., Gonzalo-Orden, J.M., and Pastor, J.C. (2013). Adalimumab (tumor necrosis factor-blocker) reduces the expression of glial fibrillary acidic protein immunoreactivity increased by exogenous tumor necrosis factor alpha in an organotypic culture of porcine neuroretina. *Mol Vis* 19**,** 894-903.

Fischer, A.J., Omar, G., Eubanks, J., McGuire, C.R., Dierks, B.D., and Reh, T.A. (2004a). Different aspects of gliosis in retinal Muller glia can be induced by CNTF, insulin, and FGF2 in the absence of damage. *Mol Vis* 10**,** 973-986.

Fischer, A.J., Schmidt, M., Omar, G., and Reh, T.A. (2004b). BMP4 and CNTF are neuroprotective and suppress damage-induced proliferation of Muller glia in the retina. *Mol Cell Neurosci* 27(4)**,** 531-542. doi: 10.1016/j.mcn.2004.08.007.

Fischer, A.J., Scott, M.A., and Tuten, W. (2009). Mitogen-activated protein kinase-signaling stimulates Muller glia to proliferate in acutely damaged chicken retina. *Glia* 57(2)**,** 166-181. doi: 10.1002/glia.20743.

Fischer, A.J., Zelinka, C., Gallina, D., Scott, M.A., and Todd, L. (2014). Reactive microglia and macrophage facilitate the formation of Muller glia-derived retinal progenitors. *Glia* 62(10)**,** 1608-1628. doi: 10.1002/glia.22703.

Fletcher, E.L. (2020). Advances in understanding the mechanisms of retinal degenerations. *Clin Exp Optom*. doi: 10.1111/cxo.13146.

Fletcher, E.L., Wang, A.Y., Jobling, A.I., Rutar, M.V., Greferath, U., Gu, B., et al. (2019). Targeting P2X7 receptors as a means for treating retinal disease. *Drug Discov Today* 24(8)**,** 1598-1605. doi: 10.1016/j.drudis.2019.03.029.

Galan, A., Jmaeff, S., Barcelona, P.F., Brahimi, F., Sarunic, M.V., and Saragovi, H.U. (2017). In retinitis pigmentosa TrkC.T1-dependent vectorial Erk activity upregulates glial TNF-alpha, causing selective neuronal death. *Cell Death Dis* 8(12)**,** 3222. doi: 10.1038/s41419-017-0074-8.

Galvao, J., Davis, B., Tilley, M., Normando, E., Duchen, M.R., and Cordeiro, M.F. (2014). Unexpected low-dose toxicity of the universal solvent DMSO. *FASEB J* 28(3)**,** 1317-1330. doi: 10.1096/fj.13-235440.

Gao, F., Li, F., Miao, Y., Xu, L.J., Zhao, Y., Li, Q., et al. (2017). Involvement of the MEK-ERK/p38-CREB/c-fos signaling pathway in Kir channel inhibition-induced rat retinal Muller cell gliosis. *Sci Rep* 7(1)**,** 1480. doi: 10.1038/s41598-017-01557-y.

Geiger, P., Barben, M., Grimm, C., and Samardzija, M. (2015). Blue light-induced retinal lesions, intraretinal vascular leakage and edema formation in the all-cone mouse retina. *Cell Death Dis* 6**,** e1985. doi: 10.1038/cddis.2015.333.

Geller, S.F., Lewis, G.P., and Fisher, S.K. (2001). FGFR1, signaling, and AP-1 expression after retinal detachment: reactive Muller and RPE cells. *Invest Ophthalmol Vis Sci* 42(6)**,** 1363-1369.

German, O.L., Insua, M.F., Gentili, C., Rotstein, N.P., and Politi, L.E. (2006). Docosahexaenoic acid prevents apoptosis of retina photoreceptors by activating the ERK/MAPK pathway. *J Neurochem* 98(5)**,** 1507-1520. doi: 10.1111/j.1471-4159.2006.04061.x.

Ghosh, F., Abdshill, H., Arner, K., Voss, U., and Taylor, L. (2018). Retinal neuroinflammatory induced neuronal degeneration - Role of toll-like receptor-4 and relationship with gliosis. *Exp Eye Res* 169**,** 99-110. doi: 10.1016/j.exer.2018.02.002.

Giaume, C., Kirchhoff, F., Matute, C., Reichenbach, A., and Verkhratsky, A. (2007). Glia: the fulcrum of brain diseases. *Cell Death Differ* 14(7)**,** 1324-1335. doi: 10.1038/sj.cdd.4402144.

Gotschel, F., Berg, D., Gruber, W., Bender, C., Eberl, M., Friedel, M., et al. (2013). Synergism between Hedgehog-GLI and EGFR signaling in Hedgehog-responsive human medulloblastoma cells induces downregulation of canonical Hedgehog-target genes and stabilized expression of GLI1. *PLoS One* 8(6)**,** e65403. doi: 10.1371/journal.pone.0065403.

Gottsch, J.D., Bynoe, L.A., Harlan, J.B., Rencs, E.V., and Green, W.R. (1993). Light-induced deposits in Bruch's membrane of protoporphyric mice. *Arch Ophthalmol* 111(1)**,** 126-129.

Gregory, M.S., Hackett, C.G., Abernathy, E.F., Lee, K.S., Saff, R.R., Hohlbaum, A.M., et al. (2011). Opposing roles for membrane bound and soluble Fas ligand in glaucoma-associated retinal ganglion cell death. *PLoS One* 6(3)**,** e17659. doi: 10.1371/journal.pone.0017659.

Grimm, C., Wenzel, A., Williams, T., Rol, P., Hafezi, F., and Reme, C. (2001). Rhodopsin-mediated blue-light damage to the rat retina: effect of photoreversal of bleaching. *Invest Ophthalmol Vis Sci* 42(2)**,** 497-505.

Groeger, G., Doonan, F., Cotter, T.G., and Donovan, M. (2012). Reactive oxygen species regulate prosurvival ERK1/2 signaling and bFGF expression in gliosis within the retina. *Invest Ophthalmol Vis Sci* 53(10)**,** 6645-6654. doi: 10.1167/iovs.12-10525.

Halasz, E., Zarbin, M.A., Davidow, A.L., Frishman, L.J., Gombkoto, P., and Townes-Anderson, E. (2021). ROCK inhibition reduces morphological and functional damage to rod synapses after retinal injury. *Sci Rep* 11(1)**,** 692. doi: 10.1038/s41598-020-80267-4.

Hauck, S.M., Kinkl, N., Deeg, C.A., Swiatek-de Lange, M., Schoffmann, S., and Ueffing, M. (2006). GDNF family ligands trigger indirect neuroprotective signaling in retinal glial cells. *Mol Cell Biol* 26(7)**,** 2746-2757. doi: 10.1128/MCB.26.7.2746-2757.2006.

Herrero-Martin, G., Hoyer-Hansen, M., Garcia-Garcia, C., Fumarola, C., Farkas, T., Lopez-Rivas, A., et al. (2009). TAK1 activates AMPK-dependent cytoprotective autophagy in TRAIL-treated epithelial cells. *EMBO J* 28(6)**,** 677-685. doi: 10.1038/emboj.2009.8.

Hollborn, M., Tenckhoff, S., Jahn, K., Iandiev, I., Biedermann, B., Schnurrbusch, U.E., et al. (2005). Changes in retinal gene expression in proliferative vitreoretinopathy: glial cell expression of HB-EGF. *Mol Vis* 11**,** 397-413.

Huang, H., Liu, Y., Wang, L., and Li, W. (2017). Age-related macular degeneration phenotypes are associated with increased tumor necrosis-alpha and subretinal immune cells in aged Cxcr5 knockout mice. *PLoS One* 12(3)**,** e0173716. doi: 10.1371/journal.pone.0173716.

Iandiev, I., Wurm, A., Hollborn, M., Wiedemann, P., Grimm, C., Reme, C.E., et al. (2008). Muller cell response to blue light injury of the rat retina. *Invest Ophthalmol Vis Sci* 49(8)**,** 3559-3567. doi: 10.1167/iovs.08-1723.

Inoue, Y., Tsuruma, K., Nakanishi, T., Oyagi, A., Ohno, Y., Otsuka, T., et al. (2013). Role of heparin-binding epidermal growth factor-like growth factor in light-induced photoreceptor degeneration in mouse retina. *Invest Ophthalmol Vis Sci* 54(6)**,** 3815-3829. doi: 10.1167/iovs.12-11236.

Jacquemin, E., de Kozak, Y., Thillaye, B., Courtois, Y., and Goureau, O. (1996). Expression of inducible nitric oxide synthase in the eye from endotoxin-induced uveitis rats. *Invest Ophthalmol Vis Sci* 37(6)**,** 1187-1196.

Janes, K.A., Gaudet, S., Albeck, J.G., Nielsen, U.B., Lauffenburger, D.A., and Sorger, P.K. (2006). The response of human epithelial cells to TNF involves an inducible autocrine cascade. *Cell* 124(6)**,** 1225-1239. doi: 10.1016/j.cell.2006.01.041.

Jia, Y., Jiang, S., Chen, C., Lu, G., Xie, Y., Sun, X., et al. (2019). Caffeic acid phenethyl ester attenuates nuclear factorkappaBmediated inflammatory responses in Muller cells and protects against retinal ganglion cell death. *Mol Med Rep* 19(6)**,** 4863-4871. doi: 10.3892/mmr.2019.10151.

Jones, B.W., Marc, R.E., and Pfeiffer, R.L. (1995). "Retinal Degeneration, Remodeling and Plasticity," in *Webvision: The Organization of the Retina and Visual System,* eds. H. Kolb, E. Fernandez & R. Nelson. (Salt Lake City (UT)).

Jones, B.W., Pfeiffer, R.L., Ferrell, W.D., Watt, C.B., Tucker, J., and Marc, R.E. (2016). Retinal Remodeling and Metabolic Alterations in Human AMD. *Front Cell Neurosci* 10**,** 103. doi: 10.3389/fncel.2016.00103.

Kanamori, A., Nakamura, M., Yamada, Y., and Negi, A. (2012). Longitudinal study of retinal nerve fiber layer thickness and ganglion cell complex in traumatic optic neuropathy. *Arch Ophthalmol* 130(8)**,** 1067-1069. doi: 10.1001/archophthalmol.2012.470.

Karl, M.O., Hayes, S., Nelson, B.R., Tan, K., Buckingham, B., and Reh, T.A. (2008). Stimulation of neural regeneration in the mouse retina. *Proc Natl Acad Sci U S A* 105(49)**,** 19508-19513. doi: 10.1073/pnas.0807453105.

Kase, S., Yoshida, K., Harada, T., Harada, C., Namekata, K., Suzuki, Y., et al. (2006). Phosphorylation of extracellular signal-regulated kinase and p27(KIP1) after retinal detachment. *Graefes Arch Clin Exp Ophthalmol* 244(3)**,** 352-358. doi: 10.1007/s00417-005-0016-5.

Kassen, S.C., Thummel, R., Campochiaro, L.A., Harding, M.J., Bennett, N.A., and Hyde, D.R. (2009). CNTF induces photoreceptor neuroprotection and Muller glial cell proliferation through two different signaling pathways in the adult zebrafish retina. *Exp Eye Res* 88(6)**,** 1051-1064. doi: 10.1016/j.exer.2009.01.007.

Kaur, S., Gupta, S., Chaudhary, M., Khursheed, M.A., Mitra, S., Kurup, A.J., et al. (2018). let-7 MicroRNA-Mediated Regulation of Shh Signaling and the Gene Regulatory Network Is Essential for Retina Regeneration. *Cell Rep* 23(5)**,** 1409-1423. doi: 10.1016/j.celrep.2018.04.002.

Kim, E.J., Grant, G.R., Bowman, A.S., Haider, N., Gudiseva, H.V., and Chavali, V.R.M. (2018). Complete Transcriptome Profiling of Normal and Age-Related Macular Degeneration Eye Tissues Reveals Dysregulation of Anti-Sense Transcription. *Sci Rep* 8(1)**,** 3040. doi: 10.1038/s41598-018-21104-7.

Kim, G.H., Kim, H.I., Paik, S.S., Jung, S.W., Kang, S., and Kim, I.B. (2016). Functional and morphological evaluation of blue light-emitting diode-induced retinal degeneration in mice. *Graefes Arch Clin Exp Ophthalmol* 254(4)**,** 705-716. doi: 10.1007/s00417-015-3258-x.

Kinkl, N., Sahel, J., and Hicks, D. (2001). Alternate FGF2-ERK1/2 signaling pathways in retinal photoreceptor and glial cells in vitro. *J Biol Chem* 276(47)**,** 43871-43878. doi: 10.1074/jbc.M105256200.

Kovacs, K., Marra, K.V., Yu, G., Wagley, S., Ma, J., Teague, G.C., et al. (2015). Angiogenic and Inflammatory Vitreous Biomarkers Associated With Increasing Levels of Retinal Ischemia. *Invest Ophthalmol Vis Sci* 56(11)**,** 6523-6530. doi: 10.1167/iovs.15-16793.

Krishnamoorthy, R.R., Crawford, M.J., Chaturvedi, M.M., Jain, S.K., Aggarwal, B.B., Al-Ubaidi, M.R., et al. (1999). Photo-oxidative stress down-modulates the activity of nuclear factor-kappaB via involvement of caspase-1, leading to apoptosis of photoreceptor cells. *J Biol Chem* 274(6)**,** 3734-3743. doi: 10.1074/jbc.274.6.3734.

Kuchroo, M., DiStasio, M., Calapkulu, E., Ige, M., Zhang, L., Sheth, A.H., et al. (2021). Topological analysis of single-cell data reveals shared glial landscape of macular degeneration and neurodegenerative diseases. *bioRxiv***,** 2021.2001.2019.427286. doi: 10.1101/2021.01.19.427286.

Kurth, T., Berger, J., Wilsch-Brauninger, M., Kretschmar, S., Cerny, R., Schwarz, H., et al. (2010). Electron microscopy of the amphibian model systems Xenopus laevis and Ambystoma mexicanum. *Methods Cell Biol* 96**,** 395-423. doi: 10.1016/S0091-679X(10)96017-2.

Kyosseva, S.V. (2016). Targeting MAPK Signaling in Age-Related Macular Degeneration. *Ophthalmol Eye Dis* 8**,** 23-30. doi: 10.4137/OED.S32200.

Laabich, A., Vissvesvaran, G.P., Lieu, K.L., Murata, K., McGinn, T.E., Manmoto, C.C., et al. (2006). Protective effect of crocin against blue light- and white light-mediated photoreceptor cell death in bovine and primate retinal primary cell culture. *Invest Ophthalmol Vis Sci* 47(7)**,** 3156-3163. doi: 10.1167/iovs.05-1621.

Lahne, M., Li, J., Marton, R.M., and Hyde, D.R. (2015). Actin-Cytoskeleton- and Rock-Mediated INM Are Required for Photoreceptor Regeneration in the Adult Zebrafish Retina. *J Neurosci* 35(47)**,** 15612-15634. doi: 10.1523/JNEUROSCI.5005-14.2015.

Lebrun-Julien, F., Duplan, L., Pernet, V., Osswald, I., Sapieha, P., Bourgeois, P., et al. (2009). Excitotoxic death of retinal neurons in vivo occurs via a non-cell-autonomous mechanism. *J Neurosci* 29(17)**,** 5536-5545. doi: 10.1523/JNEUROSCI.0831-09.2009.

Lee, H.O., Herndon, J.M., Barreiro, R., Griffith, T.S., and Ferguson, T.A. (2002). TRAIL: a mechanism of tumor surveillance in an immune privileged site. *J Immunol* 169(9)**,** 4739-4744. doi: 10.4049/jimmunol.169.9.4739.

Levine, E.M., Close, J., Fero, M., Ostrovsky, A., and Reh, T.A. (2000). p27(Kip1) regulates cell cycle withdrawal of late multipotent progenitor cells in the mammalian retina. *Dev Biol* 219(2)**,** 299-314. doi: 10.1006/dbio.2000.9622.

Li, M., Huisingh, C., Messinger, J., Dolz-Marco, R., Ferrara, D., Freund, K.B., et al. (2018). Histology of gegraphic atrophy secondary to age-related macular degeneration: A multilayer apporach. *Retina*. doi: 10.1097/IAE.0000000000002182.

Li, W., Liu, X., Tu, Y., Ding, D., Yi, Q., Sun, X., et al. (2020). Dysfunctional Nurr1 promotes high glucose-induced Muller cell activation by up-regulating the NF-kappaB/NLRP3 inflammasome axis. *Neuropeptides* 82**,** 102057. doi: 10.1016/j.npep.2020.102057.

Liddelow, S.A., Guttenplan, K.A., Clarke, L.E., Bennett, F.C., Bohlen, C.J., Schirmer, L., et al. (2017). Neurotoxic reactive astrocytes are induced by activated microglia. *Nature* 541(7638)**,** 481-487. doi: 10.1038/nature21029.

Loffler, K., Schafer, P., Volkner, M., Holdt, T., and Karl, M.O. (2015). Age-dependent Muller glia neurogenic competence in the mouse retina. *Glia* 63(10)**,** 1809-1824. doi: 10.1002/glia.22846.

Lu, W., Hu, H., Sevigny, J., Gabelt, B.T., Kaufman, P.L., Johnson, E.C., et al. (2015). Rat, mouse, and primate models of chronic glaucoma show sustained elevation of extracellular ATP and altered purinergic signaling in the posterior eye. *Invest Ophthalmol Vis Sci* 56(5)**,** 3075-3083. doi: 10.1167/iovs.14-15891.

Lyu, Y., Zauhar, R., Dana, N., Strang, C.E., Wang, K., Liu, S., et al. (2019). Integrative single-cell and bulk RNA-seq analysis in human retina identified cell type-specific composition and gene expression changes for age-related macular degeneration. *bioRxiv***,** 768143. doi: 10.1101/768143.

Martinez-Fernandez de la Camara, C., Sequedo, M.D., Gomez-Pinedo, U., Jaijo, T., Aller, E., Garcia-Tarraga, P., et al. (2013). Phosphodiesterase inhibition induces retinal degeneration, oxidative stress and inflammation in cone-enriched cultures of porcine retina. *Exp Eye Res* 111**,** 122-133. doi: 10.1016/j.exer.2013.03.015.

Matsumoto, H., Murakami, Y., Kataoka, K., Notomi, S., Mantopoulos, D., Trichonas, G., et al. (2015). Membrane-bound and soluble Fas ligands have opposite functions in photoreceptor cell death following separation from the retinal pigment epithelium. *Cell Death Dis* 6**,** e1986. doi: 10.1038/cddis.2015.334.

Medrano, M.P., Pisera-Fuster, A., Bernabeu, R.O., and Faillace, M.P. (2020). P2X7 and A2A receptor endogenous activation protects against neuronal death caused by CoCl2 -induced photoreceptor toxicity in the zebrafish retina. *J Comp Neurol* 528(12)**,** 2000-2020. doi: 10.1002/cne.24869.

Miao, X., Lv, H., Wang, B., Chen, Q., Miao, L., Su, G., et al. (2013). Deletion of angiotensin II type 1 receptor gene attenuates chronic alcohol-induced retinal ganglion cell death with preservation of VEGF expression. *Curr Eye Res* 38(1)**,** 185-193. doi: 10.3109/02713683.2012.720339.

Mitori, H., Izawa, T., Kuwamura, M., Matsumoto, M., and Yamate, J. (2016). Developing Stage-dependent Retinal Toxicity Induced by l-glutamate in Neonatal Rats. *Toxicol Pathol* 44(8)**,** 1137-1145. doi: 10.1177/0192623316676424.

Mohammad, G., AlSharif, H.M., Siddiquei, M.M., Ahmad, A., Alam, K., and Abu El-Asrar, A.M. (2018). Rho-Associated Protein Kinase-1 Mediates the Regulation of Inflammatory Markers in Diabetic Retina and in Retinal Muller Cells. *Ann Clin Lab Sci* 48(2)**,** 137-145.

Moussaud, S., and Draheim, H.J. (2010). A new method to isolate microglia from adult mice and culture them for an extended period of time. *J Neurosci Methods* 187(2)**,** 243-253. doi: 10.1016/j.jneumeth.2010.01.017.

Nagashima, M., Barthel, L.K., and Raymond, P.A. (2013). A self-renewing division of zebrafish Muller glial cells generates neuronal progenitors that require N-cadherin to regenerate retinal neurons. *Development* 140(22)**,** 4510-4521. doi: 10.1242/dev.090738.

Nakazawa, T., Kayama, M., Ryu, M., Kunikata, H., Watanabe, R., Yasuda, M., et al. (2011). Tumor necrosis factor-alpha mediates photoreceptor death in a rodent model of retinal detachment. *Invest Ophthalmol Vis Sci* 52(3)**,** 1384-1391. doi: 10.1167/iovs.10-6509.

Nakazawa, T., Matsubara, A., Noda, K., Hisatomi, T., She, H., Skondra, D., et al. (2006). Characterization of cytokine responses to retinal detachment in rats. *Mol Vis* 12**,** 867-878.

Nelson, C.M., Ackerman, K.M., O'Hayer, P., Bailey, T.J., Gorsuch, R.A., and Hyde, D.R. (2013). Tumor necrosis factor-alpha is produced by dying retinal neurons and is required for Muller glia proliferation during zebrafish retinal regeneration. *J Neurosci* 33(15)**,** 6524-6539. doi: 10.1523/JNEUROSCI.3838-12.2013.

Noailles, A., Maneu, V., Campello, L., Lax, P., and Cuenca, N. (2018). Systemic inflammation induced by lipopolysaccharide aggravates inherited retinal dystrophy. *Cell Death Dis* 9(3)**,** 350. doi: 10.1038/s41419-018-0355-x.

Olney, J.W. (1969). Glutaate-induced retinal degeneration in neonatal mice. Electron microscopy of the acutely evolving lesion. *J Neuropathol Exp Neurol* 28(3)**,** 455-474.

Olney, J.W. (1982). The toxic effects of glutamate and related compounds in the retina and the brain. *Retina* 2(4)**,** 341-359.

Palazzo, I., Deistler, K., Hoang, T.V., Blackshaw, S., and Fischer, A.J. (2020). NF-kappaB signaling regulates the formation of proliferating Muller glia-derived progenitor cells in the avian retina. *Development* 147(10). doi: 10.1242/dev.183418.

Puthussery, T., and Fletcher, E. (2009). Extracellular ATP induces retinal photoreceptor apoptosis through activation of purinoceptors in rodents. *J Comp Neurol* 513(4)**,** 430-440. doi: 10.1002/cne.21964.

Resta, V., Novelli, E., Vozzi, G., Scarpa, C., Caleo, M., Ahluwalia, A., et al. (2007). Acute retinal ganglion cell injury caused by intraocular pressure spikes is mediated by endogenous extracellular ATP. *Eur J Neurosci* 25(9)**,** 2741-2754. doi: 10.1111/j.1460-9568.2007.05528.x.

Roque, R.S., Rosales, A.A., Jingjing, L., Agarwal, N., and Al-Ubaidi, M.R. (1999). Retina-derived microglial cells induce photoreceptor cell death in vitro. *Brain Res* 836(1-2)**,** 110-119.

Saddala, M.S., Lennikov, A., Mukwaya, A., Fan, L., Hu, Z., and Huang, H. (2019). Transcriptome-wide analysis of differentially expressed chemokine receptors, SNPs, and SSRs in the age-related macular degeneration. *Hum Genomics* 13(1)**,** 15. doi: 10.1186/s40246-019-0199-1.

Sahaboglu, A., Paquet-Durand, O., Dietter, J., Dengler, K., Bernhard-Kurz, S., Ekstrom, P.A., et al. (2013). Retinitis pigmentosa: rapid neurodegeneration is governed by slow cell death mechanisms. *Cell Death Dis* 4**,** e488. doi: 10.1038/cddis.2013.12.

Samardzija, M., Corna, A., Gomez-Sintes, R., Jarboui, M.A., Armento, A., Roger, J.E., et al. (2021). HDAC inhibition ameliorates cone survival in retinitis pigmentosa mice. *Cell Death Differ* 28(4)**,** 1317-1332. doi: 10.1038/s41418-020-00653-3.

Sanchez-Lopez, A.M., Cuadros, M.A., Calvente, R., Tassi, M., Marin-Teva, J.L., and Navascues, J. (2005). Activation of immature microglia in response to stab wound in embryonic quail retina. *J Comp Neurol* 492(1)**,** 20-33. doi: 10.1002/cne.20676.

Sancho-Tello, M., Muriach, M., Barcia, J., Bosch-Morell, F., Genoves, J.M., Johnsen-Soriano, S., et al. (2008). Chronic alcohol feeding induces biochemical, histological, and functional alterations in rat retina. *Alcohol Alcohol* 43(3)**,** 254-260. doi: 10.1093/alcalc/agn006.

Sang, A., Yang, X., Chen, H., Qin, B., Zhu, M., Dai, M., et al. (2015). Upregulation of SYF2 Relates to Retinal Ganglion Cell Apoptosis and Retinal Glia Cell Proliferation After Light-Induced Retinal Damage. *J Mol Neurosci* 56(2)**,** 480-490. doi: 10.1007/s12031-015-0534-5.

Sardar Pasha, S.P.B., Munch, R., Schafer, P., Oertel, P., Sykes, A.M., Zhu, Y., et al. (2017). Retinal cell death dependent reactive proliferative gliosis in the mouse retina. *Sci Rep* 7(1)**,** 9517. doi: 10.1038/s41598-017-09743-8.

Sato, K., Li, S., Gordon, W.C., He, J., Liou, G.I., Hill, J.M., et al. (2013). Receptor interacting protein kinase-mediated necrosis contributes to cone and rod photoreceptor degeneration in the retina lacking interphotoreceptor retinoid-binding protein. *J Neurosci* 33(44)**,** 17458-17468. doi: 10.1523/JNEUROSCI.1380-13.2013.

Schafer, P., and Karl, M.O. (2017). Prospective purification and characterization of Muller glia in the mouse retina regeneration assay. *Glia* 65(5)**,** 828-847. doi: 10.1002/glia.23130.

Schindelin, J., Arganda-Carreras, I., Frise, E., Kaynig, V., Longair, M., Pietzsch, T., et al. (2012). Fiji: an open-source platform for biological-image analysis. *Nat Methods* 9(7)**,** 676-682. doi: 10.1038/nmeth.2019.

Senut, M.C., Gulati-Leekha, A., and Goldman, D. (2004). An element in the alpha1-tubulin promoter is necessary for retinal expression during optic nerve regeneration but not after eye injury in the adult zebrafish. *J Neurosci* 24(35)**,** 7663-7673. doi: 10.1523/JNEUROSCI.2281-04.2004.

Sifuentes, C.J., Kim, J.W., Swaroop, A., and Raymond, P.A. (2016). Rapid, Dynamic Activation of Muller Glial Stem Cell Responses in Zebrafish. *Invest Ophthalmol Vis Sci* 57(13)**,** 5148-5160. doi: 10.1167/iovs.16-19973.

Spence, J.R., Madhavan, M., Ewing, J.D., Jones, D.K., Lehman, B.M., and Del Rio-Tsonis, K. (2004). The hedgehog pathway is a modulator of retina regeneration. *Development* 131(18)**,** 4607-4621. doi: 10.1242/dev.01298.

Stromland, K., and Pinazo-Duran, M.D. (2002). Ophthalmic involvement in the fetal alcohol syndrome: clinical and animal model studies. *Alcohol Alcohol* 37(1)**,** 2-8.

Sudharsan, R., Beiting, D.P., Aguirre, G.D., and Beltran, W.A. (2017). Involvement of Innate Immune System in Late Stages of Inherited Photoreceptor Degeneration. *Sci Rep* 7(1)**,** 17897. doi: 10.1038/s41598-017-18236-7.

Swain, P., Kumar, S., Patel, D., Richong, S., Oberoi, P., Ghosh, M., et al. (2007). Mutations associated with retinopathies alter mitogen-activated protein kinase-induced phosphorylation of neural retina leucine-zipper. *Mol Vis* 13**,** 1114-1120.

Takeda, M., Takamiya, A., Yoshida, A., and Kiyama, H. (2002). Extracellular signal-regulated kinase activation predominantly in Muller cells of retina with endotoxin-induced uveitis. *Invest Ophthalmol Vis Sci* 43(4)**,** 907-911.

Thomas, J.L., Morgan, G.W., Dolinski, K.M., and Thummel, R. (2018). Characterization of the pleiotropic roles of Sonic Hedgehog during retinal regeneration in adult zebrafish. *Exp Eye Res* 166**,** 106-115. doi: 10.1016/j.exer.2017.10.003.

Todd, L., and Fischer, A.J. (2015). Hedgehog signaling stimulates the formation of proliferating Muller glia-derived progenitor cells in the chick retina. *Development* 142(15)**,** 2610-2622. doi: 10.1242/dev.121616.

Todd, L., Squires, N., Suarez, L., and Fischer, A.J. (2016). Jak/Stat signaling regulates the proliferation and neurogenic potential of Muller glia-derived progenitor cells in the avian retina. *Sci Rep* 6**,** 35703. doi: 10.1038/srep35703.

Todd, L., Volkov, L.I., Zelinka, C., Squires, N., and Fischer, A.J. (2015). Heparin-binding EGF-like growth factor (HB-EGF) stimulates the proliferation of Muller glia-derived progenitor cells in avian and murine retinas. *Mol Cell Neurosci* 69**,** 54-64. doi: 10.1016/j.mcn.2015.10.004.

Tonade, D., Liu, H., Palczewski, K., and Kern, T.S. (2017). Photoreceptor cells produce inflammatory products that contribute to retinal vascular permeability in a mouse model of diabetes. *Diabetologia* 60(10)**,** 2111-2120. doi: 10.1007/s00125-017-4381-5.

Townes-Anderson, E., Halasz, E., Wang, W., and Zarbin, M. (2021). Coming of Age for the Photoreceptor Synapse. *Invest Ophthalmol Vis Sci* 62(12)**,** 24. doi: 10.1167/iovs.62.12.24.

Townes-Anderson, E., Wang, J., Halasz, E., Sugino, I., Pitler, A., Whitehead, I., et al. (2017). Fasudil, a Clinically Used ROCK Inhibitor, Stabilizes Rod Photoreceptor Synapses after Retinal Detachment. *Transl Vis Sci Technol* 6(3)**,** 22. doi: 10.1167/tvst.6.3.22.

Tura, A., Schuettauf, F., Monnier, P.P., Bartz-Schmidt, K.U., and Henke-Fahle, S. (2009). Efficacy of Rho-kinase inhibition in promoting cell survival and reducing reactive gliosis in the rodent retina. *Invest Ophthalmol Vis Sci* 50(1)**,** 452-461. doi: 10.1167/iovs.08-1973.

Tyagi, P., and Santiago, C. (2018). New features in MEK retinopathy. *BMC Ophthalmol* 18(Suppl 1)**,** 221. doi: 10.1186/s12886-018-0861-8.

Ueki, Y., Karl, M.O., Sudar, S., Pollak, J., Taylor, R.J., Loeffler, K., et al. (2012). P53 is required for the developmental restriction in Muller glial proliferation in mouse retina. *Glia* 60(10)**,** 1579-1589. doi: 10.1002/glia.22377.

Ueki, Y., and Reh, T.A. (2013). EGF stimulates Muller glial proliferation via a BMP-dependent mechanism. *Glia* 61(5)**,** 778-789. doi: 10.1002/glia.22472.

Vallazza-Deschamps, G., Cia, D., Gong, J., Jellali, A., Duboc, A., Forster, V., et al. (2005). Excessive activation of cyclic nucleotide-gated channels contributes to neuronal degeneration of photoreceptors. *Eur J Neurosci* 22(5)**,** 1013-1022. doi: 10.1111/j.1460-9568.2005.04306.x.

Vazquez-Chona, F.R., Swan, A., Ferrell, W.D., Jiang, L., Baehr, W., Chien, W.M., et al. (2011). Proliferative reactive gliosis is compatible with glial metabolic support and neuronal function. *BMC Neurosci* 12**,** 98. doi: 10.1186/1471-2202-12-98.

Voigt, A.P., Whitmore, S.S., Flamme-Wiese, M.J., Riker, M.J., Wiley, L.A., Tucker, B.A., et al. (2019). Molecular characterization of foveal versus peripheral human retina by single-cell RNA sequencing. *Exp Eye Res* 184**,** 234-242. doi: 10.1016/j.exer.2019.05.001.

Volkner, M., Kurth, T., and Karl, M.O. (2019). The Mouse Retinal Organoid Trisection Recipe: Efficient Generation of 3D Retinal Tissue from Mouse Embryonic Stem Cells. *Methods Mol Biol* 1834**,** 119-141. doi: 10.1007/978-1-4939-8669-9_9.

Volkner, M., Kurth, T., Schor, J., Ebner, L.J.A., Bardtke, L., Kavak, C., et al. (2021). Mouse Retinal Organoid Growth and Maintenance in Longer-Term Culture. *Front Cell Dev Biol* 9**,** 645704. doi: 10.3389/fcell.2021.645704.

Volkner, M., Wagner, F., Steinheuer, L.M., Carido, M., Kurth, T., Yazbeck, A., et al. (2022). HBEGF-TNF induce a complex outer retinal pathology with photoreceptor cell extrusion in human organoids. *Nat Commun* 13(1)**,** 6183. doi: 10.1038/s41467-022-33848-y.

Wan, J., Ramachandran, R., and Goldman, D. (2012). HB-EGF is necessary and sufficient for Muller glia dedifferentiation and retina regeneration. *Dev Cell* 22(2)**,** 334-347. doi: 10.1016/j.devcel.2011.11.020.

Wan, J., Zhao, X.F., Vojtek, A., and Goldman, D. (2014). Retinal injury, growth factors, and cytokines converge on beta-catenin and pStat3 signaling to stimulate retina regeneration. *Cell Rep* 9(1)**,** 285-297. doi: 10.1016/j.celrep.2014.08.048.

Wan, J., Zheng, H., Xiao, H.L., She, Z.J., and Zhou, G.M. (2007). Sonic hedgehog promotes stem-cell potential of Muller glia in the mammalian retina. *Biochem Biophys Res Commun* 363(2)**,** 347-354. doi: 10.1016/j.bbrc.2007.08.178.

Wang, J., Zhang, N., Beuve, A., and Townes-Anderson, E. (2012). Mislocalized opsin and cAMP signaling: a mechanism for sprouting by rod cells in retinal degeneration. *Invest Ophthalmol Vis Sci* 53(10)**,** 6355-6369. doi: 10.1167/iovs.12-10180.

Wang, T., Tsang, S.H., and Chen, J. (2017). Two pathways of rod photoreceptor cell death induced by elevated cGMP. *Hum Mol Genet* 26(12)**,** 2299-2306. doi: 10.1093/hmg/ddx121.

Wang, W., Halasz, E., and Townes-Anderson, E. (2019). Actin Dynamics, Regulated by RhoA-LIMK-Cofilin Signaling, Mediates Rod Photoreceptor Axonal Retraction After Retinal Injury. *Invest Ophthalmol Vis Sci* 60(6)**,** 2274-2285. doi: 10.1167/iovs.18-26077.

Wen, R., Song, Y., Cheng, T., Matthes, M.T., Yasumura, D., LaVail, M.M., et al. (1995). Injury-induced upregulation of bFGF and CNTF mRNAS in the rat retina. *J Neurosci* 15(11)**,** 7377-7385.

Wen, R., Tao, W., Li, Y., and Sieving, P.A. (2012). CNTF and retina. *Prog Retin Eye Res* 31(2)**,** 136-151. doi: 10.1016/j.preteyeres.2011.11.005.

White, D.T., Sengupta, S., Saxena, M.T., Xu, Q., Hanes, J., Ding, D., et al. (2017). Immunomodulation-accelerated neuronal regeneration following selective rod photoreceptor cell ablation in the zebrafish retina. *Proc Natl Acad Sci U S A* 114(18)**,** E3719-E3728. doi: 10.1073/pnas.1617721114.

Wu, T., Chen, Y., Chiang, S.K., and Tso, M.O. (2002). NF-kappaB activation in light-induced retinal degeneration in a mouse model. *Invest Ophthalmol Vis Sci* 43(9)**,** 2834-2840.

Xia, H., Hu, Q., Li, L., Tang, X., Zou, J., Huang, L., et al. (2019). Protective effects of autophagy against blue light-induced retinal degeneration in aged mice. *Sci China Life Sci* 62(2)**,** 244-256. doi: 10.1007/s11427-018-9357-y.

Xie, J., Zhu, R., Peng, Y., Gao, W., Du, J., Zhao, L., et al. (2017). Tumor necrosis factor-alpha regulates photoreceptor cell autophagy after retinal detachment. *Sci Rep* 7(1)**,** 17108. doi: 10.1038/s41598-017-17400-3.

Xin, H., Yannazzo, J.A., Duncan, R.S., Gregg, E.V., Singh, M., and Koulen, P. (2007). A novel organotypic culture model of the postnatal mouse retina allows the study of glutamate-mediated excitotoxicity. *J Neurosci Methods* 159(1)**,** 35-42. doi: 10.1016/j.jneumeth.2006.06.013.

Xue, W., Cojocaru, R.I., Dudley, V.J., Brooks, M., Swaroop, A., and Sarthy, V.P. (2011). Ciliary neurotrophic factor induces genes associated with inflammation and gliosis in the retina: a gene profiling study of flow-sorted, Muller cells. *PLoS One* 6(5)**,** e20326. doi: 10.1371/journal.pone.0020326.

Yang, L.P., Zhu, X.A., and Tso, M.O. (2007). Role of NF-kappaB and MAPKs in light-induced photoreceptor apoptosis. *Invest Ophthalmol Vis Sci* 48(10)**,** 4766-4776. doi: 10.1167/iovs.06-0871.

Yoshida, K., Kase, S., Nakayama, K., Nagahama, H., Harada, T., Ikeda, H., et al. (2004). Distribution of p27(KIP1), cyclin D1, and proliferating cell nuclear antigen after retinal detachment. *Graefes Arch Clin Exp Ophthalmol* 242(5)**,** 437-441. doi: 10.1007/s00417-004-0861-7.

Yuan, L., and Neufeld, A.H. (2000). Tumor necrosis factor-alpha: a potentially neurodestructive cytokine produced by glia in the human glaucomatous optic nerve head. *Glia* 32(1)**,** 42-50.

Zencak, D., Schouwey, K., Chen, D., Ekstrom, P., Tanger, E., Bremner, R., et al. (2013). Retinal degeneration depends on Bmi1 function and reactivation of cell cycle proteins. *Proc Natl Acad Sci U S A* 110(7)**,** E593-601. doi: 10.1073/pnas.1108297110.

Zhang, T., Wei, Y., Jiang, X., Li, J., Qiu, S., and Zhang, S. (2015). Protection of photoreceptors by intravitreal injection of the Y-27632 Rho-associated protein kinase inhibitor in Royal College of Surgeons rats. *Mol Med Rep* 12(3)**,** 3655-3661. doi: 10.3892/mmr.2015.3889.

Zhao, L., Zabel, M.K., Wang, X., Ma, W., Shah, P., Fariss, R.N., et al. (2015). Microglial phagocytosis of living photoreceptors contributes to inherited retinal degeneration. *EMBO Mol Med* 7(9)**,** 1179-1197. doi: 10.15252/emmm.201505298.
