# Supplementary figures and images for: Modeling inducible neuropathologies of the retina with differential phenotypes in organoids
Source: Front Cell Neurosci. 2023 May 5;17:1106287. doi: 10.3389/fncel.2023.1106287 (PMC10196395; doi:10.3389/fncel.2023.1106287)

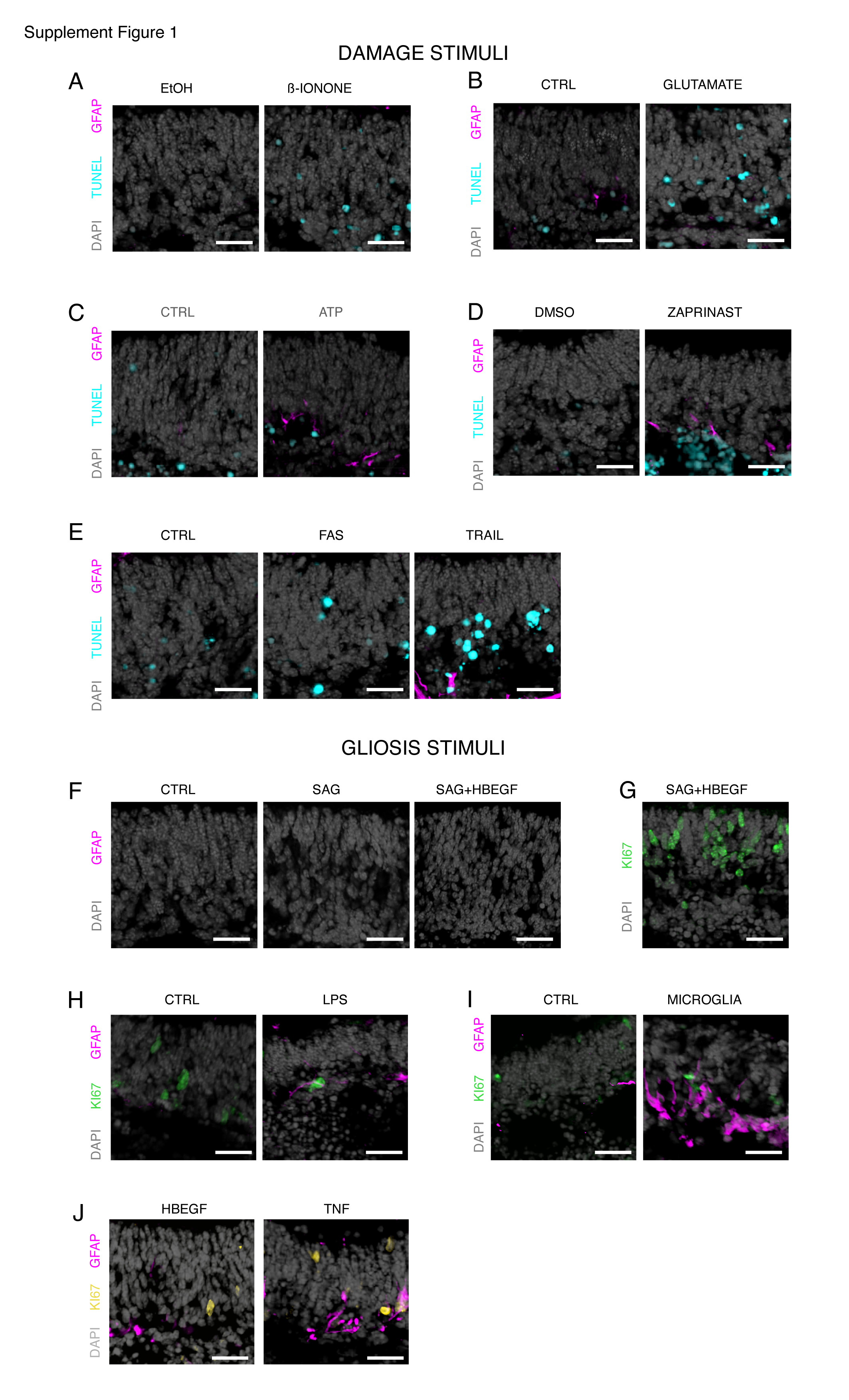

Supplement: Supplementary file 2 [file Image_1.JPEG]

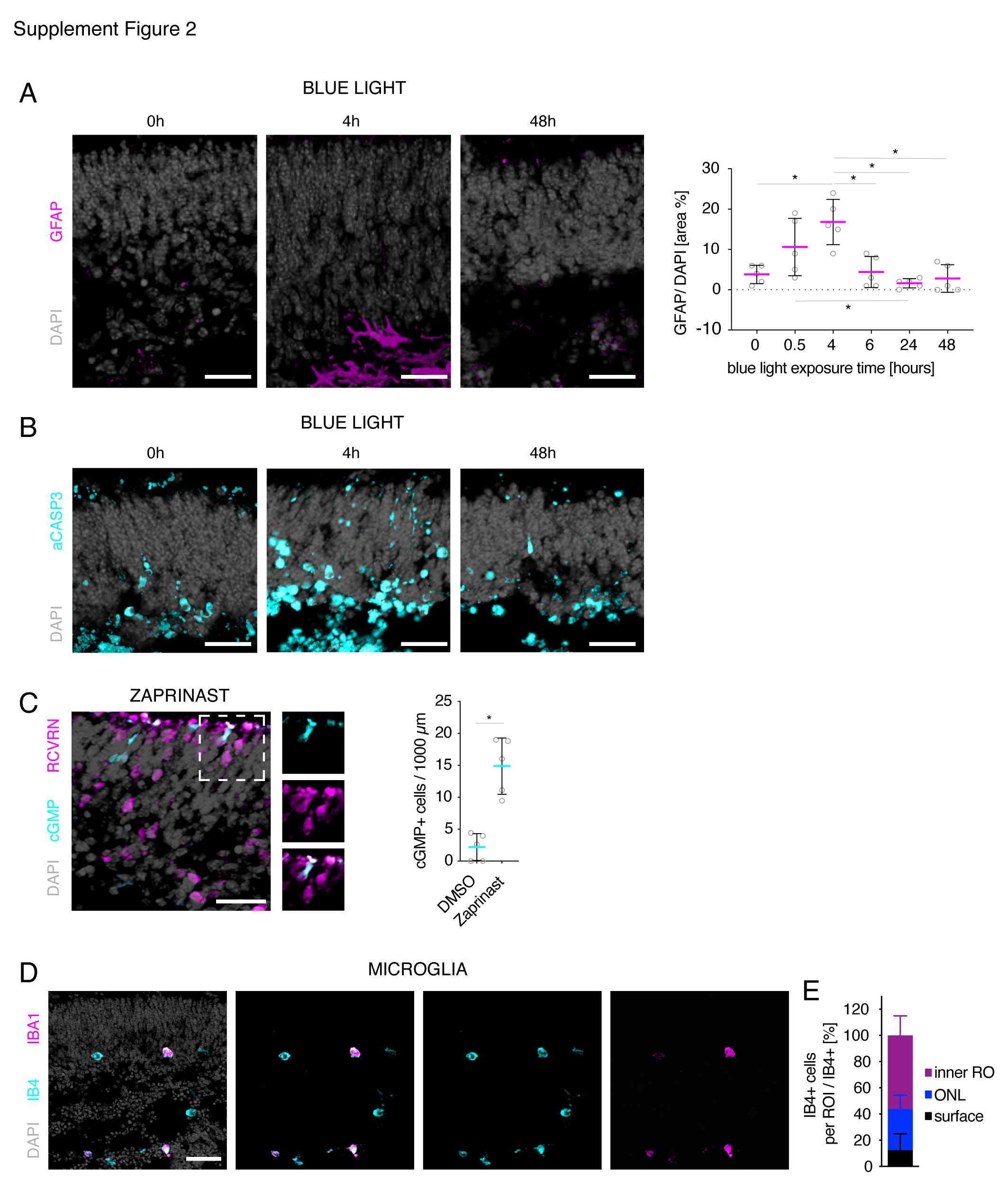

Supplement: Supplementary file 3 [file Image_2.JPEG]

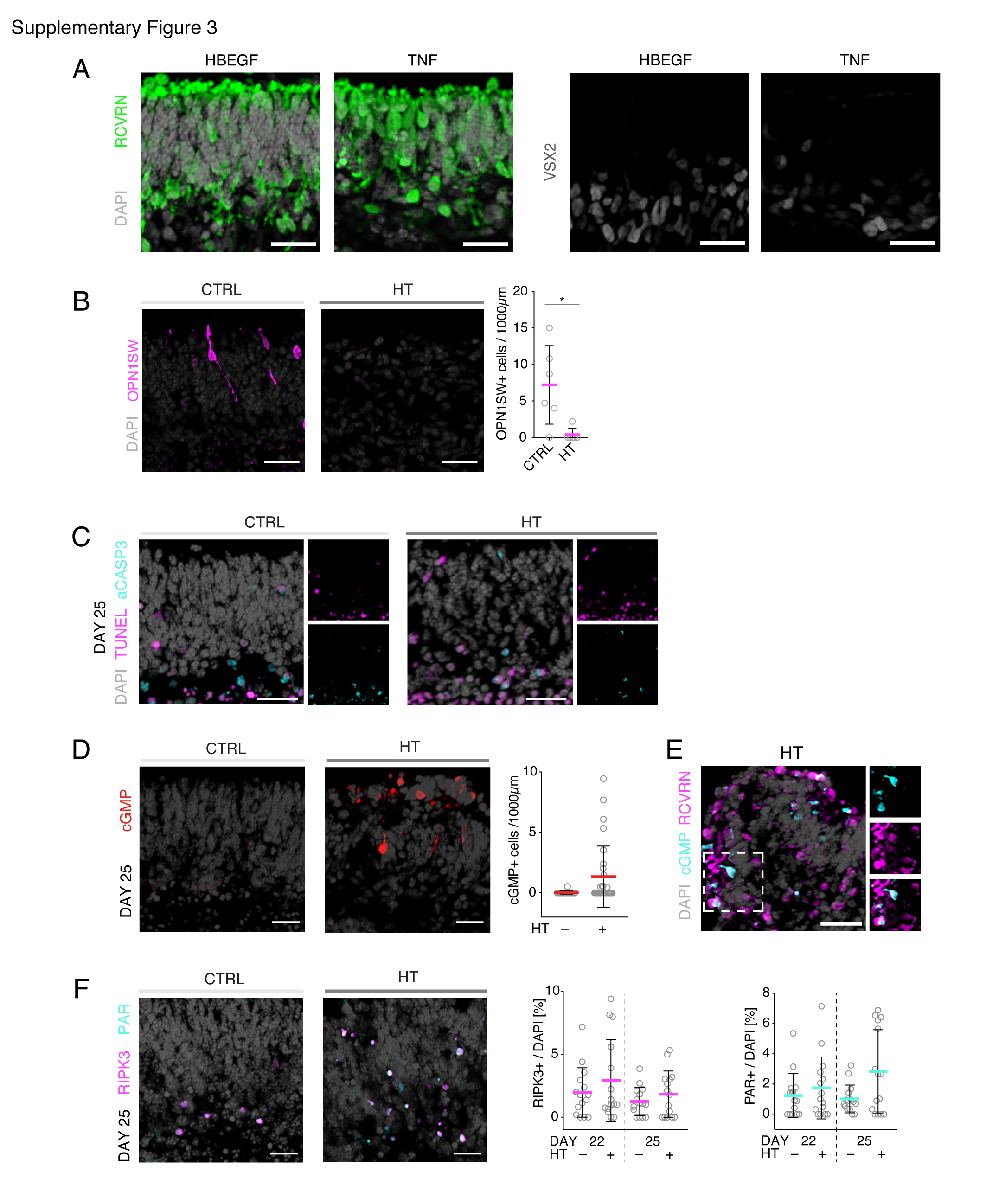

Supplement: Supplementary file 4 [file Image_3.JPEG]

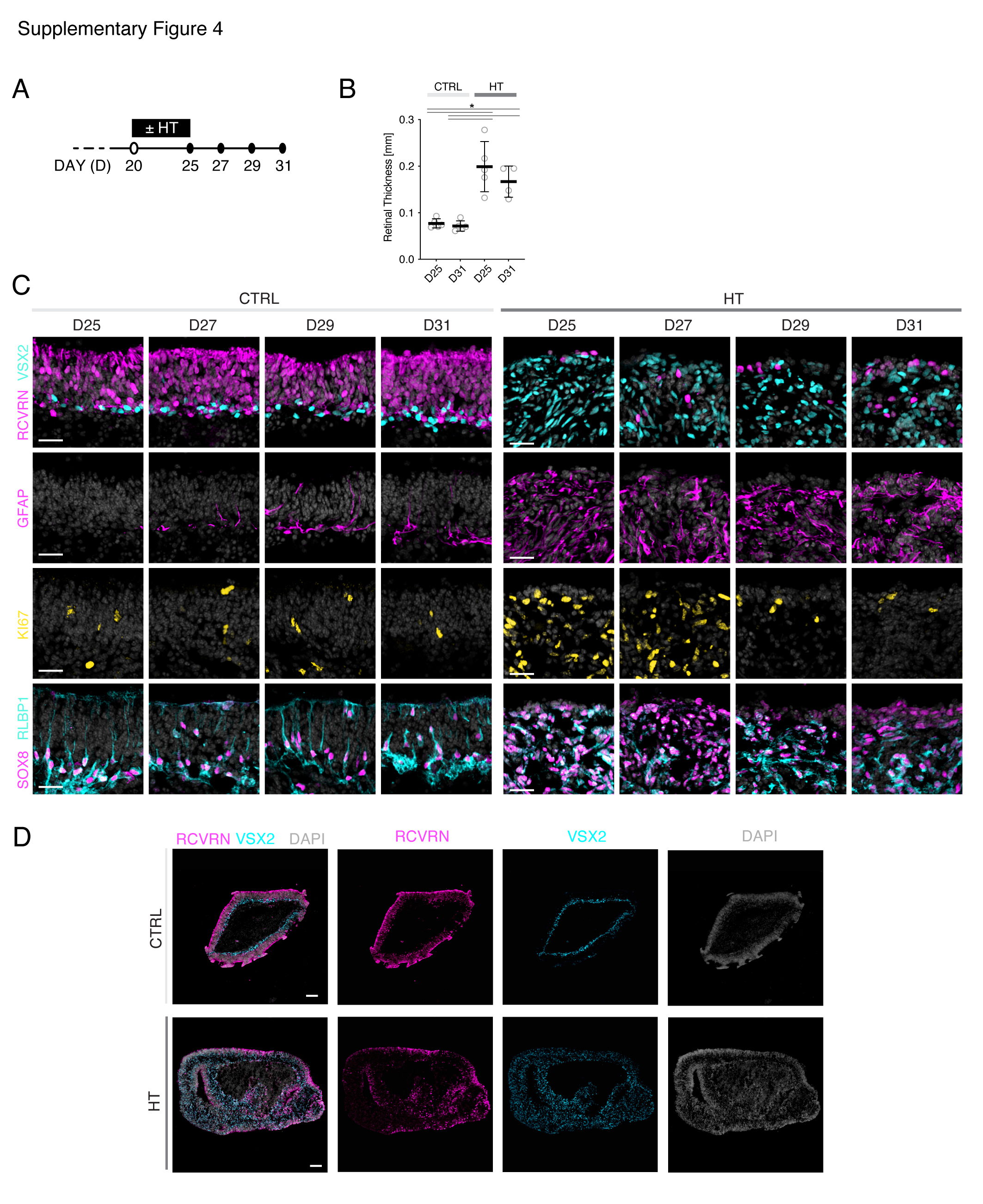

Supplement: Supplementary file 5 [file Image_4.JPEG]

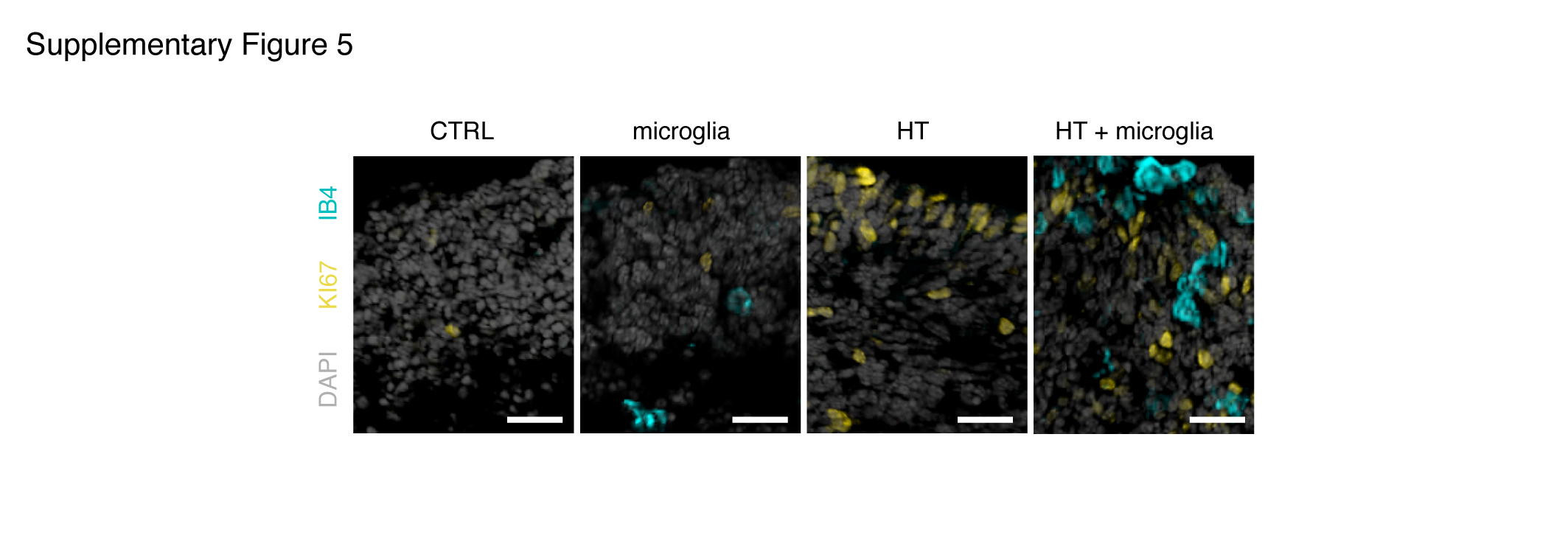

Supplement: Supplementary file 6 [file Image_5.JPEG]

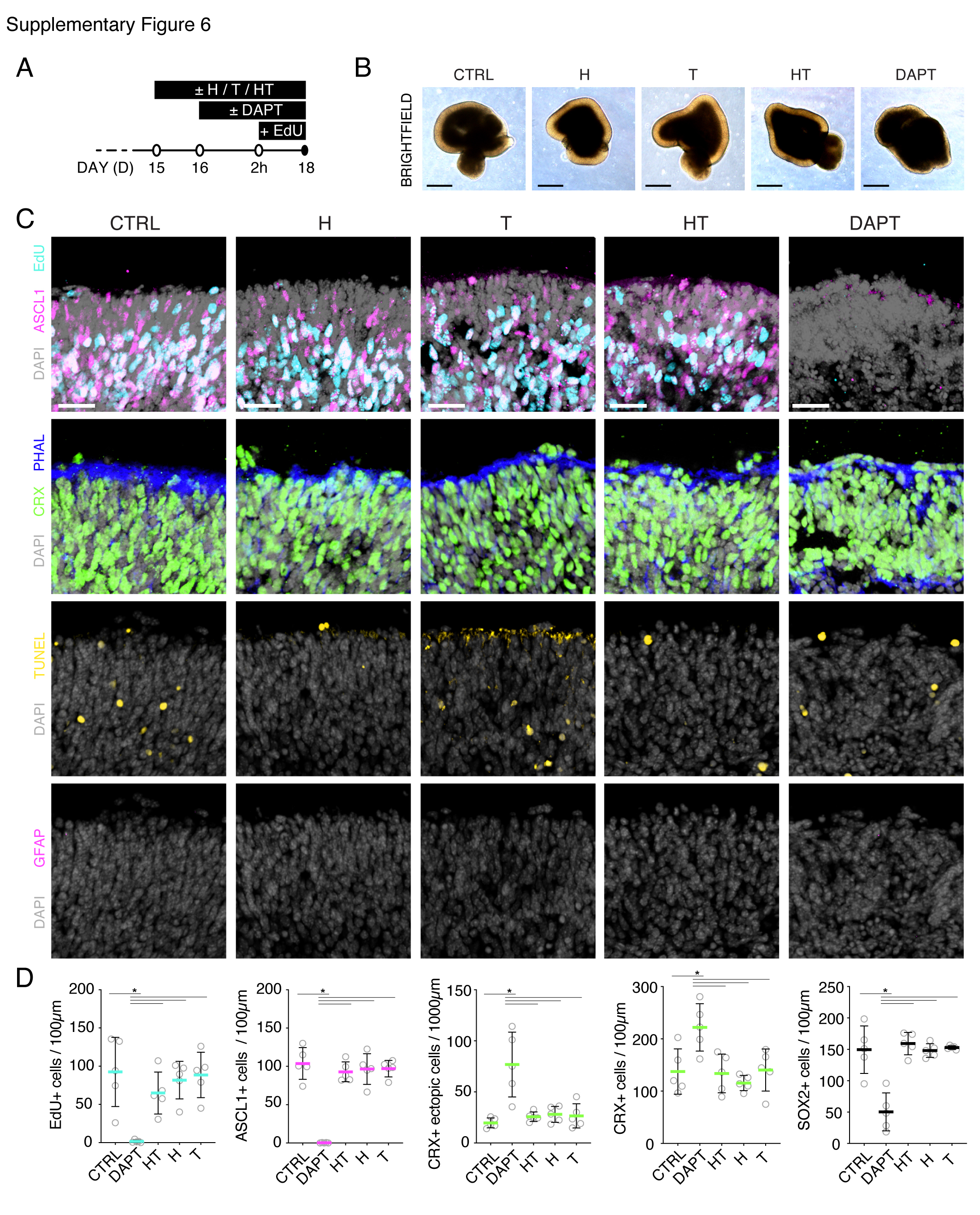

Supplement: Supplementary file 7 [file Image_6.JPEG]

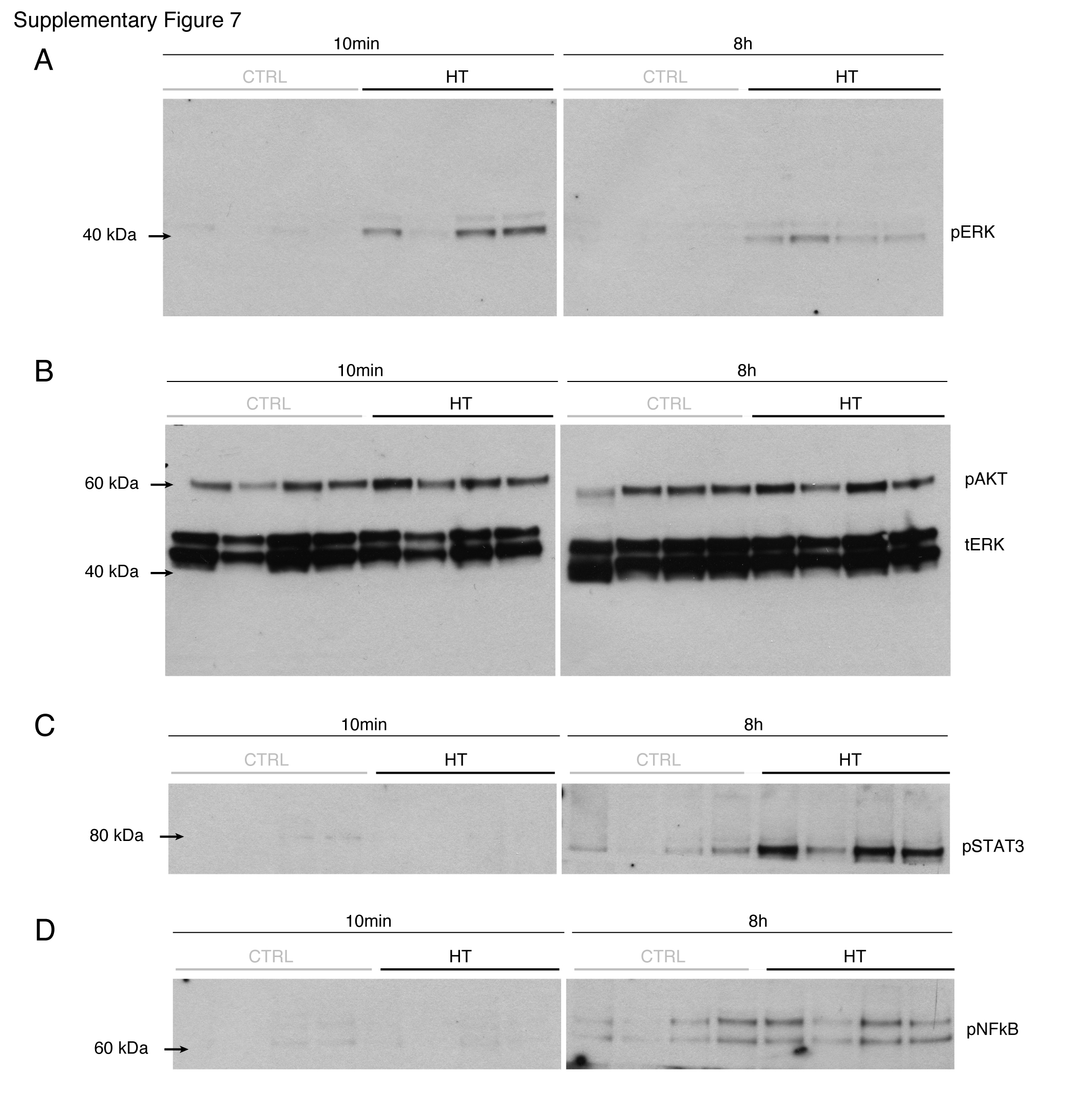

Supplement: Supplementary file 8 [file Image_7.JPEG]
